# Disrupting quorum sensing as a strategy to inhibit bacterial virulence in human, animal, and plant pathogens

**DOI:** 10.1093/femspd/ftae009

**Published:** 2024-05-09

**Authors:** Mélanie Gonzales, Baptiste Kergaravat, Pauline Jacquet, Raphaël Billot, Damien Grizard, Éric Chabrière, Laure Plener, David Daudé

**Affiliations:** Aix Marseille University, MEPHI, IHU-Méditerranée Infection, 19-21 Boulevard Jean Moulin, Marseille 13005, France; Gene&GreenTK, 19-21 Boulevard Jean Moulin, Marseille 13005, France; Aix Marseille University, MEPHI, IHU-Méditerranée Infection, 19-21 Boulevard Jean Moulin, Marseille 13005, France; Gene&GreenTK, 19-21 Boulevard Jean Moulin, Marseille 13005, France; Gene&GreenTK, 19-21 Boulevard Jean Moulin, Marseille 13005, France; Gene&GreenTK, 19-21 Boulevard Jean Moulin, Marseille 13005, France; Gene&GreenTK, 19-21 Boulevard Jean Moulin, Marseille 13005, France; Aix Marseille University, MEPHI, IHU-Méditerranée Infection, 19-21 Boulevard Jean Moulin, Marseille 13005, France; Gene&GreenTK, 19-21 Boulevard Jean Moulin, Marseille 13005, France; Gene&GreenTK, 19-21 Boulevard Jean Moulin, Marseille 13005, France

**Keywords:** quorum sensing, bacteria, virulence, biofilm, lactonase, One Health

## Abstract

The development of sustainable alternatives to conventional antimicrobials is needed to address bacterial virulence while avoiding selecting resistant strains in a variety of fields, including human, animal, and plant health. Quorum sensing (QS), a bacterial communication system involved in noxious bacterial phenotypes such as virulence, motility, and biofilm formation, is of utmost interest. In this study, we harnessed the potential of the lactonase *Sso*Pox to disrupt QS of human, fish, and plant pathogens. Lactonase treatment significantly alters phenotypes including biofilm formation, motility, and infection capacity. In plant pathogens, *Sso*Pox decreased the production of plant cell wall degrading enzymes in *Pectobacterium carotovorum* and reduced the maceration of onions infected by *Burkholderia glumae*. In human pathogens, lactonase treatment significantly reduced biofilm formation in *Acinetobacter baumannii, Burkholderia cepacia*, and *Pseudomonas aeruginosa*, with the cytotoxicity of the latter being reduced by *Sso*Pox treatment. In fish pathogens, lactonase treatment inhibited biofilm formation and bioluminescence in *Vibrio harveyi* and affected QS regulation in *Aeromonas salmonicida*. QS inhibition can thus be used to largely impact the virulence of bacterial pathogens and would constitute a global and sustainable approach for public, crop, and livestock health in line with the expectations of the One Health initiative.

## Teaser

Disrupting quorum sensing as a promising alternative to conventional antimicrobials as part of the One Health initiative.

## Introduction

The rise of antimicrobial resistance (AMR) has led the international community to reinforce hygiene measures to limit the spread of infections. Priorities include reducing the use of antimicrobials, which is a major issue, as their excessive use in livestock, human health, and agriculture induces serious AMR concerns worldwide. In this context, the World Health Organization (WHO) has initiated a “Global Action Plan” to tackle AMR (GAP-AMR) (Harbarth et al. 2015, World Health Organization 2015). Developing sustainable and global approaches to reducing bacterial infections with neither toxic side effects nor associated resistance is extremely challenging. Moving from conventional chemical approaches to novel bio-based alternatives is thus an attractive prospect. Bacteriophages, antimicrobial peptides, biological control, and enzymes are appealing candidates for a variety of applications (Nigam et al. 2014, Khardori et al. 2020). Enzymes in particular may be of interest, as their catalytic performances may allow them to be used in small quantities while having a broad effect. Nevertheless, to limit the emergence of AMR, rethinking how to inhibit bacterial virulence remains a major concern. Over the last two decades, enzymes able to disrupt quorum sensing (QS) have gained considerable interest with regards to limiting bacterial infections while not challenging bacterial survival (Rémy et al. 2016). QS is a bacterial communication process relying on the production and detection of autoinducible signaling molecules (AIs) that bacteria use to coordinate behaviors in a cell-density-dependent manner, including infection-related behaviors. AIs are chemically diverse, including acyl-homoserine lactones (AHLs), furanosyl diester AI-2, peptides, and hormones (e.g. epinephrine) that can be recognized by one or several bacteria depending on the signal (Rémy et al. 2018). Among AIs, special attention has been paid to AHLs, as they are widely used by Gram-negative bacteria, including human, animal, and plant pathogens. Enzymes such as lactonases, acylases, and oxidoreductases have been studied for their ability to interfere with AHL-based QS, a strategy referred to as quorum quenching (QQ). AHLs are signaling molecules presenting a conserved lactone ring and an acyl chain moiety that may vary in length (from C_4_ to C_18_), substitutions (e.g. hydroxyl and carbonyl), and degree of saturation. Basically, AHL-based QS relies on a synthase, usually noted “I,” that produces one or several AHLs that diffuse freely through cell membranes, and a sensor regulator, noted “R” which, upon AHL binding, becomes active and induces the expression of numerous genes that often include the synthase I, thus generating autoinduction (Papenfort and Bassler 2016). As a broad panel of AHLs is involved in QS regulation, QQ enzymes (QQE) require large substrate acceptance in order to efficiently target numerous bacteria. Here, we aimed to evaluate *Sso*Pox lactonase, a hyperstable enzyme of great interest for biotechnological applications, on a wide panel of AHL-using bacteria. Our objective was to probe the potential of lactonase technology to deal with bacterial threats in different fields of application. As the development of an alternative to antibiotics and conventional antimicrobials is challenging, this study aims to demonstrate that the same lactonase, and its related variants, can be used as a global alternative in a range of important applications. To this end, four variants of the lactonase *Sso*Pox, obtained by semirational engineering strategies, were considered on the basis of their distinct specificities toward AHLs (Billot et al. 2022, Daude et al. 2023). As all bacteria do not necessarily use the same AHLs for their respective QS, a wide panel of pathogenic bacteria involved in plant infections (*Burkholderia glumae, Dickeya chrysanthemi, Dickeya dadantii*, and *Pectobacterium carotovorum*), human infections (*Acinetobacter baumannii, Burkholderia cepacia, Pseudomonas aeruginosa*), and animal infections (*Aeromonas salmonicida* and *Vibrio harveyi*) was selected. The ability of these strains to produce AHLs was checked by the identification of an AHL synthase in their respective genomes (Table 1). The chosen enzymatic variants showed different abilities to interfere with pathogenic bacteria. Nevertheless, each strain was sensitive to at least one variant, resulting in alteration of QS-related behaviors in these strains. These results suggest that enzyme-mediated QQ, combining one or more variants, may be considered as a multipurpose approach to strengthening the antimicrobial arsenal, as promoted by the GAP-AMR. Lactonases could thus constitute a sustainable approach to address health threats in the animal–human–environmental interface in line with the One Health initiative (McEwen and Collignon 2018, One Health 2024).

**Table 1.** *Sso*Pox variants and concentration used for each strain.

Strains	Culture medium	*Sso*Pox variants	Concentration (mg ml^−1^)
*A. baumannii*	LB	A	0.1
*A. salmonicida*	TSB	A	0.5
*B. cepacia*	SSM	B	0.09
*B. glumae*	LB	B	0.09
*D. chrysanthemi*	TSB	C	0.16
*D. dadantii*	TSB	C	0.16
*P. carotovorum* (DSM)	TSB	D	0.055
*P. carotovorum* (Vegepolys)	TSB	D	0.055
*P. aeruginosa*	MOPS	A	0.1
*V. harveyi*	AB	C and D	0.5 for both

## Material and methods

### Strains and bacterial cultures

The following strains were used in this study: *A. baumannii* ATCC 17978, *A. salmonicida* ATCC 33658, *B. cepacia* DSM 7288 (ATCC 25416), *B. glumae* DSM 9512, *Chromobacterium violaceum* CV026, *D. chrysanthemi* DV1910-2771 (Vegepolys field isolate from carrot), *D. dadantii* DSM 18020, *P. carotovorum* DSM 30168 (from the German Collection of Microorganisms and Cell Cultures (DSMZ)), *P. carotovorum* DV1708-1686 (Vegepolys field isolate from tomato), *P. aeruginosa* PA14, and *V. harveyi* DSM 19623. All the strains were first precultivated from a single colony in Lysogeny Broth (LB: 10 g l^−1^ NaCl, 10 g l^−1^ tryptone, 5 g l^−1^ yeast extract, and pH 7), except for *D. dadantii, D. chrysanthemi*, and both *P. carotovorum*, which were precultivated in Tryptic Soy Broth (TSB). A culture step was then performed as indicated below, before the different phenotypic assays. *Chromobacterium violaceum* CV026 was cultivated in LB at 37°C and used as a reporter strain for the detection of AHLs.

Regarding phytopathogenic bacteria, the inoculation of *B. glumae* from a 16-hour preculture was carried out at 1:100 dilution in fresh LB. Subsequently, 3 ml of this culture was poured into 12-well plates and was then incubated for 24 hours at 33°C, with stirring at 350 rpm. The cultures of *D. dadantii, D. chrysanthemi*, and both *P. carotovorum* from the DSMZ and Vegepolys collections, were performed from a 6-hour preculture diluted at 1:100 in TSB. A quantity of 1.5 ml of the culture was transferred to 24-well plates and then incubated for 16 hours at 30°C, with agitation at 400 rpm.

For the strains related to human health, *A. baumannii* was inoculated at a 1:100 dilution in fresh LB from a 16-hour preculture. Then, 3 ml of the inoculated culture was poured into 12-well plates and incubated for 48 hours at 37°C under static conditions. *Burkholderia cepacia* was inoculated at 1:1000 from a 16-hour preculture in standard succinate media (Meyer 1978) (6 g l^−1^ K_2_HPO_4_, 3 g l^−1^ KH_2_PO_4_, 0.2 g l^−1^ MgSO_4_.7H_2_O, 1 g l^−1^ [NH_4_]_2_SO_4_, 0.4 g l^−1^ acid succinic, and pH was adjusted to 7 with NaOH). A quantity of 2 ml of culture was poured into 24-well plates and incubated for 24 hours at 37°C, with stirring (350 rpm). For *P. aeruginosa*, a 6-hour preculture was diluted 1:1000 in MOPS minimal medium, as described previously (Rémy et al. 2020) (50 mM MOPS, 4 mM Tricine, 50 mM NaCl, 1 mM K_2_SO_4_, 50 µM MgCl_2_, 10 µM CaCl_2_, 0.3 µM (NH_4_)_6_Mo_7_O_24_, 40 µM H_3_BO_3_, 3 µM Co(OAc)_2_, 1 µM CuSO_4_, 8 µM MnSO_4_, 1 µM ZnSO_4_, and adjusted to pH 7 and filter sterilized), complemented with nitrogen (15 mM NH_4_Cl), iron (5 µM Fe_2_SO_4_), phosphate (4 mM K_2_HPO_4_), and carbon (25 mM glutamate) sources. A quantity of 3 ml of culture was added to 12-well plates and incubated for 24 hours at 37°C with a 350 rpm agitation.

Finally, for the veterinary strains, cultures of *A. salmonicida* were performed from a 16-hour preculture and diluted at 1:1000 in TSB. Cultures were grown for 24 hours at room temperature without stirring in a 24-well plate. *Vibrio harveyi* was precultivated in LB for 16 hours and inoculated 1:100 in autoinducer bioassay (AB) medium (Greenberg et al. 1979) [0.3 M NaCl, 0.05 M MgSO_4_, and 0.2% vitamin-free casamino acids (Difco), adjusted to pH 7.5 with KOH and supplemented with 200 µl of sterile 1 M potassium phosphate (pH 7.0), 200 µl of 0.1 M l-arginine, and 250 µl of glycerol 80% for a final volume of 20 ml]. The cultures were transferred to 24-well plates and incubated for 24 hours at 30°C with stirring (350 rpm).

*Sso*Pox variants were added during the cultures, and their respective concentrations were adjusted according to their specific kinetic parameters. The variant and the concentration used for each strain are indicated in Table 1.

### Production of SsoPox enzymes and determination of their concentration

Enzyme production was performed as described previously (Hiblot et al. 2013). Briefly, *Escherichia coli* BL21 (DE3)-pGro7/GroEL strain containing the plasmid of the *Sso*Pox variant (W263I, V82I-A266G-A275Y, V82I-A275G, and V82I-K271L) was used. Variants W263I, V82I-A266G-A275Y, V82I-A275G, and V82I-K271 L are hereafter referred to as A, B, C, and D, respectively. Precultures were incubated at 37°C for 16 hours in LB supplemented with antibiotics (ampicillin at 100 µg ml^−1^ and chloramphenicol at 34 µg ml^−1^). Bacteria were then inoculated at 1:100 in ZYP-5052 autoinducer medium supplemented with both antibiotics described above and incubated at 37°C with 180 rpm agitation. When cultures reached an OD 600 nm between 0.8 and 1, bacteria were induced by adding 0.2% l-arabinose for chaperon production and CoCl_2_ at 0.2 mM final concentration, to stabilize the active site of *Sso*Pox lactonase. The temperature was reduced to 23°C and cultures were grown for 20 hours with stirring at 180 rpm. Cells were harvested by centrifugation at 4 000 × *g* for 20 minutes at 10°C. Pellets were solubilized in lysis buffer (HEPES 50 mM pH 8, NaCl 150 mM, DNAseI 10 µg ml^−1^, lysozyme 0.25 mg ml^− 1^, and PMSF 0.1 mM). Cells in the lysis buffer were stored at −80°C for at least 16 hours. Thawed cells were sonicated three times for 30 seconds, with an amplitude of 45% (QSonica sonicator Q700 sonicator®, QSonica, USA). Finally, cells were heated to 80°C for 30 minutes and then centrifuged to pellet the debris. The supernatants were collected and saturated with 75% of ammonium sulfate at 4°C for 16 hours to precipitate *Sso*Pox. The enzyme was pelleted down by centrifugation at 10 000 × *g* for 15 minutes at 10°C. The pellets were resuspended in 8 ml of HEPES 50 mM pH 8.0 and NaCl 150 mM buffer and then filtered at 0.8 µm. Ammonium sulfate was removed via desalting (HiPrep 26/10 desalting, ÄKTA pure, GE Healthcare, USA ÄKTA pure). The sample was concentrated using 30 kDa centricon, and then injected into a size-exclusion chromatography column (GF Hiload 16/600 Superdex 75 pg) to purify the enzyme. Enzyme purity was then checked by SDS-PAGE and protein concentration was measured using a Bradford assay (Bradford 1976).

### Measurement of lactonase activity

Kinetic parameters were determined as previously described (Rémy et al. 2020), by following the degradation of lactones (C_4_-HSL, 3-OH-C_4_-HSL, C_6_-HSL, 3-oxo-C_6_-HSL, C_8_-HSL, 3-oxo-C_8_-HSL, C_10_-HSL, 3-oxo-C_10_-HSL, C_12_-HSL, and 3-oxo-C_12_-HSL) at different concentrations (in a range of concentrations between 0 and 2 mM), by each enzymatic variant in a cresol buffered solution (1.25 mM Bicine, 150 mM NaCl, 0.2 mM cresol purple, 10% of DMSO were supplemented for C_10_-HSL, and pH was adjusted to 8.3). The degradation of AHLs was followed for 10 minutes at 577 nm in 200 µl using a plate reader (SynergyHT, BioTek). Mean values were fitted to the Michaelis–Menten equation using GraphPad Prism 7.04 software to obtain catalytic parameters.

### Detection of AHL

Six-well plates containing 3 ml of LB agar supplemented or not supplemented with enzyme, were prepared to identify AHL production. A quantity of 10 µl of bacterial culture was streaked in line on the agar next to another line of 10 µl of the reporter strain *C. violaceum* CV026 from a 16-hour preculture in LB. This strain produces violacein when exogenous AHLs are detected (McClean et al. 1997). *Sso*Pox action on QS was evaluated by the abolition of violacein production upon degradation of AHL produced by the tested strains. Plates were incubated at room temperature for 48 hours to observe violacein production by CV026.

### Assessment of biofilm formation

Biofilm formation was measured for each strain using the crystal violet staining method (Sigma®) (Hoffman et al. 2005). Inoculation for each strain was performed as detailed above in the section “Strains and bacterial cultures.” After incubation for 16 hours (*P. carotovorum* and *Dickeya* sp.), 24 hours (*A. salmonicida, Burkholderia* sp., *P. aeruginosa*, and *V. harveyi*) and 48 hours (*A. baumannii*) at the appropriate temperatures, planktonic cells were carefully removed from the 12- or 24-well plates. Wells were washed with phosphate buffered saline (PBS) and completely dried at 37°C. Then, 3 ml (for 12-well plates) and 2 ml (for 24-well plates) of 0.05% crystal violet was added to stain the attached biofilm, and each plate was stained for 10 minutes. Crystal violet was gently removed, and the wells were washed with PBS. The fixed crystal violet was finally dissolved in 3 or 2 ml of absolute ethanol, depending on the plate, and 200 µl was transferred to a 96-well plate. Biofilm formation was quantified by measuring the absorbance at OD 595 nm in a microplate reader. The following classification was applied for the determination of biofilm formation: Absorbances below 0.3 indicate the absence of an attached biofilm, absorbances between 0.3 and 1 reveal a weak biofilm formation, and absorbances above 3 are evidence of a strong biofilm (Xu et al. 2016).

### Measurement of plant cell wall degrading enzymes

Plant cell wall degrading enzymes (PCWDE) assays were performed by measuring the activity of pectate lyase, polygalacturonase, and cellulase.

The pectate lyase plates were prepared according to a previously reported procedure (Starr et al. 1977). Briefly, the plates were composed of 1% (w/v) polygalacturonic acid, 1% (w/v) yeast extract, 10% (v/v) 0.1% bromothymol blue, and 1.5% agar in distilled water. The solution was heated, without boiling, until all the components were dissolved, and the pH was adjusted to 7. If necessary, the pH was readjusted after autoclave sterilization. Supernatants from overnight cultures treated or not treated with *Sso*Pox were harvested, and 10 µl of the supernatants was added to the plates and incubated for 4 hours at 37°C. Polygalacturonic acid degradation halos were revealed by pouring 4 N HCl on the plates to precipitate nondegraded polygalacturonic acid. Pectate lyase activity in the supernatants was assessed by the presence of clear halos in the dish.

Polygalacturonase activity measurement was performed as follows (Cui et al. 2020). Briefly, plates were poured with a mixture of 0.5% polygalacturonic acid, 0.2% sucrose, 0.2% (NH_4_)_2_SO_4_, and 1.5% agar. A quantity of 10 µl of cell-free supernatants was spotted on the plates. After incubation for 4 hours at 37°C, the plates were revealed with 4 N HCl. The formation of halos indicated the degradation of polygalacturonic acid by polygalacturonase in the supernatants.

Finally, the detection of cellulase activity was measured, as previously described (Cui et al. 2020). Plates were prepared with 0.1% carboxymethyl cellulose, 0.8% agarose and 0.38% sodium phosphate, and pH was adjusted to 7. A quantity of 10 µl of supernatants was spotted on the plate and after 4 hours of incubation at 37°C the plates were revealed with 5 minutes incubation at room temperature with 0.1% Congo red, followed by two washes with 1 M NaCl. Carboxymethyl cellulose degradation was detected by the formation of a white halo, indicating the presence of cellulase activity in the supernatants.

### Swarming motility assay

Swarming motility was monitored for *B. glumae*. Briefly, 3 µl from a 24-hour culture was spotted on 0.7% LB agar supplemented or not supplemented with *Sso*Pox B. 24-well plates were incubated at 30°C for 48 hours, maintaining a high humidity atmosphere to avoid agarose drying. Motility areas were measured using ImageJ. Experiments were performed in four replicates.

### Virulence assay on onion slices

Red onions (*Allium cepa*), purchased from a local market, were used in this assay to assess the virulence of *B. glumae*. The onions were disinfected for 15 minutes in a 70% ethanol bath, and thoroughly rinsed with sterile water. A sterile knife was used to cut the onions into six pieces. The slices were separated and perforated with tips prior to infection. Bacteria were centrifuged at 10 000 × *g* for 10 minutes and supernatants were removed.

The onions were then infected with 10 µl of culture adjusted to 0.25 × 10^8^ CFU ml^−1^ with PBS. To evaluate the effect of *Sso*Pox B on *B. glumae* virulence, when indicated, onion slices were pretreated with 0.09 mg ml^−1^ of lactonase and left to dry. The onion slices were then infected with bacterial culture. PBS was inoculated as negative control. The onion slices were placed on Petri dishes on a filter paper soaked with 3 ml of sterile water and placed in a box containing paper towel moistened with 30 ml of distilled water to maintain high humidity. The onion slices were incubated in the dark at 30°C for 5 days (Karki et al. 2012). Three onions were used in this assay, and 15 slices were included in each condition.

To quantitatively assess virulence, the macerated tissues were removed using a spatula and the percentage of macerated tissues was quantified by weighing the slices before and after removing maceration (Carstens et al. 2018).

### Cytotoxicity of *P. aeruginosa* toward J774.1 macrophages

J774.1 murine macrophages were used to evaluate the cytotoxicity of *P. aeruginosa*. These macrophages were cultivated in Roswell Park Memorial Institute (RPMI) 1640 broth (Gibco™, Thermo Fisher Scientific, Waltham, MA, USA) supplemented with 10% foetal bovine serum (FBS) and 1% streptomycin and penicillin (Gibco^™^, Thermo Fisher Scientific) and incubated at 37°C in a humidified atmosphere containing 5% CO_2_. When macrophages reached 75% confluence, cells were harvested in a 50-ml tube and centrifuged for 5 minutes at 750 × *g* and 4°C. Cells were resuspended in RPMI supplemented with 10% FBS to remove the antibiotics, and then centrifuged again. Finally, cells were placed in a 96-well plate at 10^5^ cells per well in 100 µl RPMI with 10% FBS. Cells were incubated for 20 hours at 37°C in a humidified atmosphere containing 5% CO_2_.

*Pseudomonas aeruginosa*, cultivated as described above, was centrifuged and the pellet was diluted in RPMI broth supplemented with 10% FBS. A quantity of 10 µl of bacteria was added to J774.1 macrophages at a multiplicity of infection (MOI) of 10. Supernatant toxicity was also evaluated by adding 10 µl to the J774.1 macrophages. Centrifugation (10 minutes, 500 × *g*, 4°C) was performed to bring bacteria into contact with the macrophages, before incubating the 96-well plates for 45 minutes at 37°C with 5% CO_2_. Finally, supernatants were collected after a 5-minute centrifugation step at 200 × *g* and 4°C.

Cytotoxicity was assessed by a CyQUANT™ lactate dehydrogenase (LDH) cytotoxicity assay kit (Thermo Fisher Scientific) according to the manufacturer’s instructions, by measuring LDH released by macrophages damaged by bacteria. The negative control was measured by adding PBS, corresponding to 0% cytotoxicity, and the positive control by adding the commercial lysis buffer, corresponding to 100% toxicity, to noninfected macrophage cells.

### RNA extraction and QS gene expression measurement

*Aeromonas salmonicida* was grown as previously described, with or without the addition of *Sso*Pox enzyme. A volume of 200 µl of culture was used for RNA extraction with the RNA PureLink® mini kit (Thermo Fisher). After extraction, a DNase treatment was performed with the TURBO DNA-free^TM^ kit (Thermo Fisher) to eliminate DNA contamination. Reverse transcription was then performed using the Taqman® Reverse Transcription reagents kit (Thermo Fisher) and random hexamers to obtain cDNA. Finally, qPCR was performed with LightCycler® SYBR® Green I Master (Roche) in CFX thermocycler (BioRad). The primers used are indicated in Table 2. As a control gene, *recA* was used and *asaIR* were targeted as *luxIR* homologs (Swift et al. 1997). qPCR data were analysed using the comparative Ct method (Tinh et al. 2007, Schmittgen and Livak 2008, Pande et al. 2013), to calculate 2^−ΔCt^. Statistical analyses were performed with GraphPad Prism 8 software, multiple *t*-test analyses were used with statistical significance of 0.05. The experiment was performed twice, with three replicates each time (*n* = 6).

**Table 2.** Sequences of primers used for RT-qPCR experiments.

Primers	*A. salmonicida*
*recA*	Fwd: ATTGCGGAAGCCCAGAAGAA
	Rev: GTATCCGGCTGGGAGATCAG
*asaI*	Fwd: ATGGGAGGTAGAAAACGAGCTT
	Rev: GGCCTTCTTCGTCCTCGAT
*asaR*	Fwd: ACCAACTGCTTGAGTACCTCG
	Rev: TGATCAGTGCGAACCGGTAG

## Results and discussion

### Selection of *Sso*Pox candidates for a broad QQ potential study

The importance of QQE specificity to efficiently tackle QS-regulated bacterial virulence has been thoroughly demonstrated (Koch et al. 2014, Rémy et al. 2020), and reviewed (Murugayah and Gerth 2019, Billot et al. 2020, Sikdar and Elias 2020). These publications underlined that the right QQE may disrupt a precise QS signal with maximum efficacy. Protein engineering strategies were implemented, particularly on the highly robust *Sso*Pox, leading to drastic changes in activities toward AHLs (Bzdrenga et al. 2017, Billot et al. 2020). Hence, to demonstrate the potential of the QQ strategy toward a wide range of pathogens relying on different QS systems, four *Sso*Pox variants, generated by our group (Hiblot et al. 2013, Billot et al. 2022, Daude et al. 2023), with distinct specificities on AHL substrates, were carefully selected and tested from a collection of previously mutated enzymes. Briefly, the collection was rationally designed through site saturation mutagenesis targeting residues involved in substrate binding and active site flexibility, leading to catalytic activity modulation toward AHLs (Hiblot et al. 2013, Billot et al. 2022). Here, four variants were selected from laboratory screenings, referred to as *Sso*Pox A–D (A: W263I, B: V82I-A266G-A275Y, C: V82I-A275G, and D: V82I-K271L), and were thoroughly characterized (Table 3), revealing (i) improved degradation activities toward several AHLs and (ii) distinct and complementary specificities towards different types of AHLs. *Sso*Pox A was selected for its high catalytic activity towards long-chain AHLs such as 3-oxo-C_12_-HSL, with a k_cat_/K_M_ > 10^5^ M^−1^ s^−1^ (Hiblot et al. 2013). This enzyme has already been shown to efficiently tackle the QS-related virulence of various pathogens, notably several strains of *P. aeruginosa* (Hraiech et al. 2014, Guendouze et al. 2017, Rémy et al. 2020), and *C. violaceum* (Mion et al. 2021). This enzyme thus constitutes a good candidate to target pathogens using long-chain AHLs. *Sso*Pox B is a multiple variant with improved specificity towards AHLs carrying a 6-carbon acyl chain, such as C_6_-HSL and 3-oxo-C_6_-HSL (10^4^ M^−1^ s^−1^ and 10^3^ M^−1^ s^−1^, respectively). This variant is the best candidate so far to target these AHLs. Similarly, variant *Sso*Pox D was selected for its broader specificity towards mid-range AHLs, such a C_6_, C_8_, and C_10_ acyl chain AHLs. This enzyme could be qualified as broad-range, since it is the only one to reach a k_cat_/K_M_ value > 10^3^ M^−1^ s^−1^ on six different AHLs, and up to 10^4^ M^−1^ s^−1^ towards C_8_-HSL and 3-oxo-C_10_-HSL. Finally, *Sso*Pox C was the variant selected to disrupt QS systems relying on short-chain AHLs, such as C_4_-HSL. While its catalytic efficiency toward C_4_-HSL remains relatively low (10^3^ M^−1^ s^−1^), it is the best activity reported so far for an *Sso*Pox variant, the wild-type enzyme being almost inactive towards this substrate (11.62 M^−1^ s^−1^) (Hiblot et al. 2013). Furthermore, its efficiency at disrupting the QS-regulated phenotypes of a C_4_-HSL model bacterium has been previously established (Daude et al. 2023).

**Table 3.** Kinetic parameters of different *Sso*Pox variants.

Enzyme	Kinetic parameter	C_4_	C_6_	3-oxo-C_6_	C_8_	3-oxo-C_8_	C_10_	3-oxo-C_10_	3-oxo-C_12_
**A**	k_cat_ (s^-1^)	0.37 ± 0.01^ǂ^	0.46 ± 0.02^¤^	0.18 ± 0.03^¤^	1.03 ± 0.03	0.85 ± 0.04^¤^	0.90 ± 0.13$	0.60 ± 0.09^□^	1.80 ± 0.05^□^
	K_M_ (µM)	4.3 (± 0.1) × 10^3^^ǂ^	9.99 (± 0.72) × 10^2^^¤^	2.81 (± 0.69) × 10^4^^¤^	1.79 (± 0. 22) × 10^2^	8.74 (± 0.84) × 10^2^^¤^	6.64 (± 2.08) × 10^2^$	1.61 (± 0.44) × 10^3^^□^	1.78 (± 0.49) × 10^3^^□^
	k_cat_/K_M_ (s^-1^.M^-1^)	8.6 (± 0.1) × 10^1^^ǂ^	4.58 (± 0.48) × 10^2^^¤^	4.34 (± 1.79) × 10^1^^¤^	5.75 (± 0.90) × 10^3^	9.69 (± 1.35) × 10^2^^¤^	1.35 (± 0.60) × 10^2^$	3.74 (± 1.17) × 10^2^^□^	**1.01 (± 0.28) × 10^5^** ^□^
**B**	k_cat_ (s^-1^)	0.37 ± 0.04^⁑^	6.70 ± 0.17^⁑^	2.23 ± 0.11^⁑^	2.05 ± 0.11	1.15 ± 0.04	N.D	0.20 ± 0.01	0.08 ± 0.01
	K_M_ (µM)	9.54 (±0.20) × 10^2^^⁑^	2.77 (± 0.27) × 10^2^^⁑^	9.40 (± 1.06) × 10^2^^⁑^	4.21 (± 0.61) × 10^2^	6.72 (± 0.62) × 10^2^		2.01 (± 0.30) × 10^2^	6.51 (± 1.80) × 10^1^
	k_cat_/K_M_ (s^-1^.M^-1^)	3.92 (± 1.17) × 10^2^^⁑^	**2.42 (±0.30) × 10^4^** ^⁑^	**2.37 (± 0.39) × 10^3^** ^⁑^	4.86 (±0.96) × 10^3^	1.70 (± 0.22) × 10^3^		9.84 (± 1.96) × 10^2^	1.25 (± 0.42) × 10^3^
**C**	k_cat_ (s^-1^)	2.13 ± 0.08^⁑^	8.42 ± 0.03^⁑^	2.24 ± 0.11^⁑^	1.74 ± 0.13	6.78 ± 1.12	N.D	1.79 ± 0.11	5.62 (± 0.23) × 10^–2^
	K_M_ (µM)	6.17 (± 0.68) × 10^2^^⁑^	1.06 (±0.01) × 10^4^^⁑^	2.49 (± 0.18) × 10^4^^⁑^	1.37 (± 0.18) × 10^3^	3.57 (± 0.89) × 10^3^		6.84 (± 0.81) × 10^2^	1.15 (± 0.18) × 10^2^
	k_cat_/K_M_ (s^-1^.M^-1^)	**3.45 (± 0.51) × 10^3^** ^⁑^	7.96 (±0.63) × 10^3^^⁑^	9.02 (± 1.08) × 10^2^^⁑^	1.27 (± 0.25) × 10^3^	1.89 (± 0.30) × 10^3^		2.61 (± 0.47) × 10^3^	4.90 (± 0.96) × 10^2^
**D**	k_cat_ (s^-1^)	0.75 ± 0.02^⁑^	8.48 ± 0.14^⁑^	1.29 ± 0.06^⁑^	4.49 ± 0.26	1.85 (± 0.33) × 10^1^	0.45 ± 0.02	3.00 ± 0.18	0.25 ± 0.011
	K_M_ (µM)	1.42 (± 0.07) × 10^3^^⁑^	3.55 (±0.20) × 10^2^^⁑^	9.06 (± 0.84) × 10^2^^⁑^	3.07 (± 0.62) × 10^2^	2.10 (± 0.62) × 10^3^	8.52 (± 2.05) × 10^1^	2.08 (± 0.54) × 10^2^	5.22 (± 0.65) × 10^2^
	k_cat_/K_M_ (s^-1^.M^-1^)	2.86 (± 0.22) × 10^2^^⁑^	2.39 (±0.17) × 10^4^^⁑^	1.43 (± 0.20) × 10^3^^⁑^	**1.46 (± 0.38) × 10^4^**	**8.80 (± 3.06) × 10^3^**	5.25 (± 1.47) × 10^3^	**1.44 (± 0.46) × 10^4^**	4.69 (± 0.79) × 10^2^

ǂ from Rémy et al. (2020).

¤ from Billot et al. (2022).

□ from Hiblot et al. (2013).

⁑ from Billot et al. (unpublished).

^$^from Mion et al. (2021), N.D: not detected.

Taken as a whole, these four variants harbour complementary activity spectra, making it possible to tackle heterogeneous QS systems using different kind of AHLs more accurately and specifically. Hence, this narrow selection provides a sound basis for targeting the QS-regulated virulence phenotypes of a large spectrum of microorganisms causing deleterious issues in human health, fish farming, and food crops.

### *Sso*Pox inhibits virulence factors in phytopathogens

Many bacterial strains are of critical interest in agriculture and ornamental plant production, due to their pathogenicity. In particular, *Dickeya* and *Pectobacterium* are soft rot-inducing bacteria, present in the top 10 phytopathogenic bacteria (Mansfield et al. 2012), damaging a wide variety of crops (Charkowski 2018). Other bacterial species are also known to be noxious to specific cultures, for instance *B. glumae* to rice (Ham et al. 2011). Here, the ability of *Sso*Pox lactonase to disrupt QS of strains from three relevant genera of phytopathogenic bacteria was evaluated.

**Figure 1.**
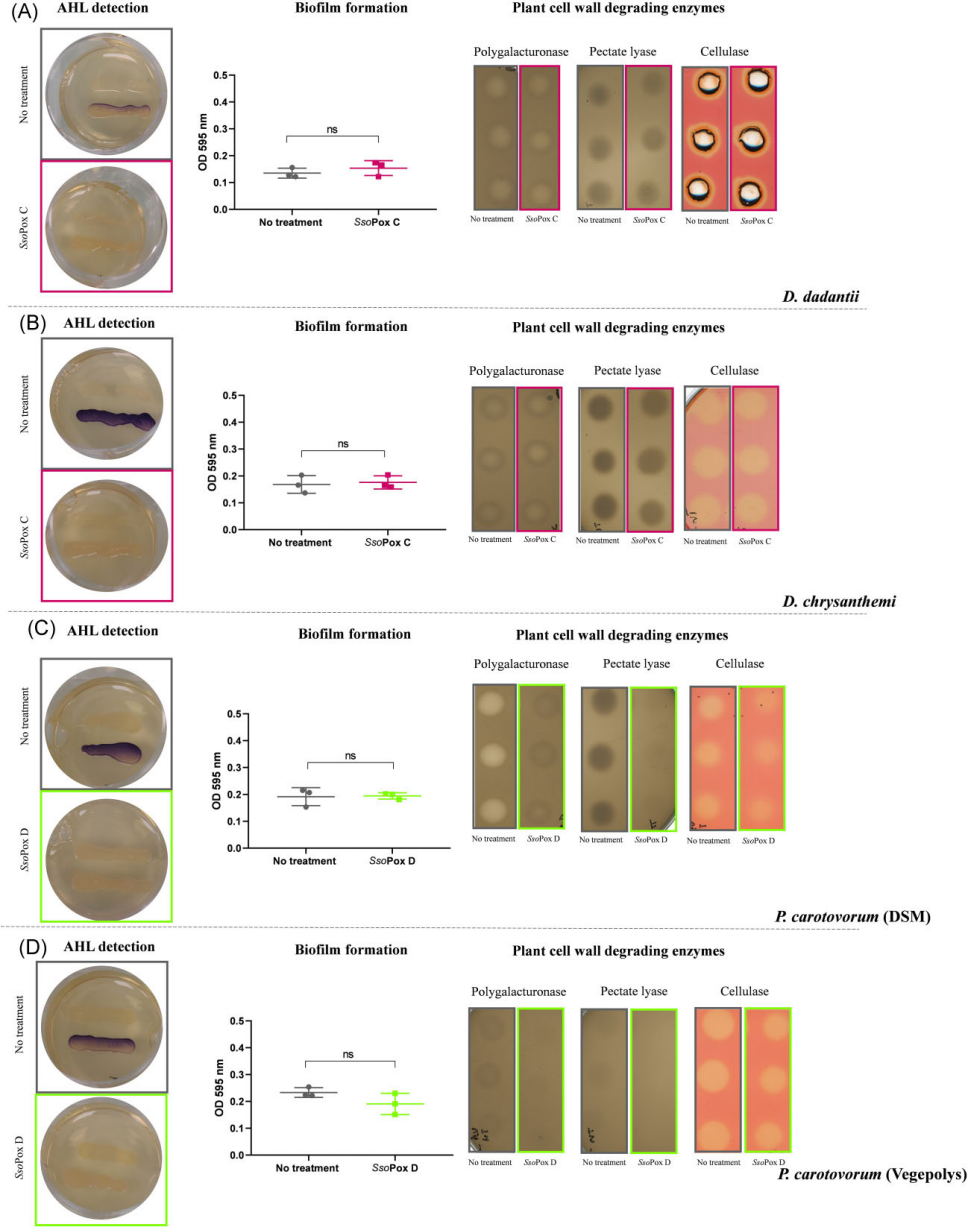
The effect of *Sso*Pox variants on four phytopathogenic strains. Detection of AHL production using CV026, measurement of biofilm formation with crystal violet staining, and impact of *Sso*Pox enzymes on PCWDE on (A) *D. dadantii*, (B) *D. chrysanthemi*, (C) *P. carotovorum* (DSM), and (D) *P. carotovorum* (Vegepolys). Statistical analysis was performed using nonparametric Mann–Whitney test, ns: nonsignificant, on GraphPad Prism 7.04.

Previous studies reported various AHL levels and types for *Dickeya sp*. and *P. carotovotum spp*., such as 3-oxo-C_6_-HSL, 3-oxo-C_8_-HSL, C_6_-HSL, and C_10_-HSL for the former, and 3-oxo-C_6_-HSL, 3-oxo-C_8_-HSL, and C_8_-HSL for the latter (Crépin et al. 2012). In this respect, *Sso*Pox C and *Sso*Pox D were selected to perform QQ experiments (Table 3). The production of AHLs was checked for each selected phytopathogenic bacterium using the reporter strain *C. violaceum* CV026 (McClean et al. 1997). The production of AHLs was detected for each strain, leading to different levels of violacein production (Fig. 1). In the presence of *Sso*Pox variants, no violacein was produced, indicating that the enzyme effectively degraded the AHLs produced by the phytopathogenic bacteria (Fig. 1).

The production of PCWDEs is a common bacterial virulence factor (Toth et al. 2003) that can be under AHL-mediated QS control (Barnard et al. 2007, Liu et al. 2008). Therefore, to evaluate QQ efficiency, semiquantitative assays were used to detect the activity of three PCWDEs, polygalacturonase, pectate lyase, and cellulase, in the supernatants from cultures of *D. dadantii, D. chrysanthemi, P. carotovorum* DMSZ30168, and *P. carotovorum* DV1708-1686 (Fig. 1).

The three PCWDE activities were detected in culture supernatants of both *Dickeya* strains, regardless of treatment with *Sso*Pox lactonase, indicating that QQ is not sufficient to abolish the expression of PCWDEs in these strains (Fig. 1A and B). In addition to AHL-mediated QS, *Dickeya* sp. are known to use another signal known as virulence factor modulating (Vfm), which has been shown to be involved in PCWDE production in some strains (Nasser et al. 2013, Helman and Chernin 2015). However, in other *Dickeya* species, such as *D. chrysanthemi*, expression of the *pel* gene was shown to be under the control of C_10_-HSL (Hosseinzadeh et al. 2017). The interplay between the two QS systems, AHLs and Vfm, has not been fully described and may be strain-dependent. Here, we showed that AHL degradation did not lead to a decrease in PCWDE production, suggesting that another signal dominates the regulation of this phenotype.

In *Pectobacterium* sp., the three enzymatic activities pectate lyase, polygalacturonase, and cellulase were decreased in the culture supernatants after lactonase treatment (Fig. 1C and D). Specifically, pectate lyase and polygalacturonase activities dramatically decreased, while cellulase activity was only slightly reduced. This result is consistent with previous studies, which demonstrated that AHL-mediated QS regulates PCWDE in *Pectobacterium*, relying on gene invalidation and recombinant lactonase expression experiments (Dong et al. 2001, Smadja et al. 2004, Palmer et al. 2011, Kusada et al. 2017). However, this is the first demonstration that PCWDE production by *Pectobacterium* sp. can be quenched by the exogenous addition of a purified lactonase.

To extend these assays, biofilm formation, another commonly studied virulence factor, was assessed for all *Dickeya* and *Pectobacterium* strains. In previous studies, biofilm and pellicle production by *Dickeya* and *Pectobacterium* was shown to vary between strains and culture conditions (Mee-Ngan et al. 2005, Joshi et al. 2015). In the conditions tested, no biofilm production was obtained and therefore no difference between treatments was observed.

**Figure 2.**
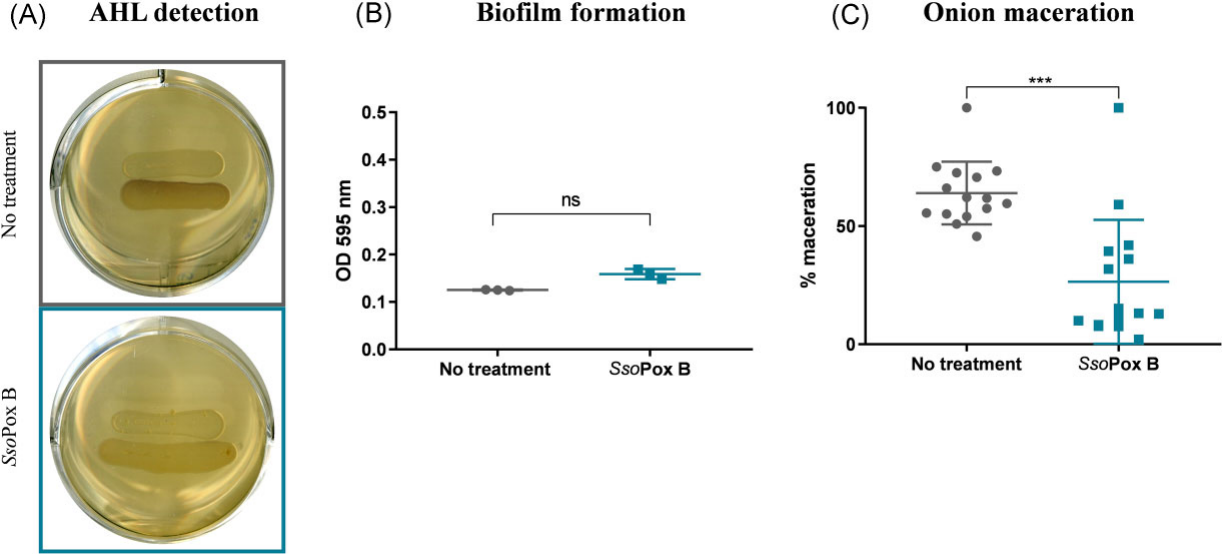
*Sso*Pox B alters *B. glumae* phenotypes. (A) Detection of AHL production using CV026. (B) Measurement of biofilm formation with crystal violet staining. Statistical difference was measured according to Mann–Whitney test on GraphPad Prism 7.04, ns: nonsignificant (C) Onion maceration, *n* = 15 slices for each condition. ***P-value < .001 according to Mann–Whitney test on GraphPad Prism 7.04.

Another phytopathogenic bacterium tested was *B. glumae*, a Gram-negative rice pathogen causing bacterial panicle blight and grain rot (Shew et al. 2019, Choi et al. 2021). This bacterium is responsible for up to 75% of rice production losses in several producer countries, including Japan (Shew et al. 2019, Cui et al. 2021). Due to global warming, *B. glumae* infections could become more common and regular over the coming decades (Shew et al. 2019), especially due to the range of its optimal growth temperature (from 30°C to 35°C) (Mizobuchi et al. 2020). In addition to rice pathogenicity, *B. glumae* is involved in wilting symptoms in sunflowers, tomatoes, sesame, perilla, eggplants, and hot peppers (Jeong et al. 2003), and of rot in onions (Karki et al. 2012). *B. glumae* has been described to produce C_8_-HSL through the TofIR QS system (Kim et al. 2004). The production of AHLs was evaluated using the reporter strain CV026. Violacein production was observed in untreated samples. Conversely, no violacein production was observed upon supplementation with *Sso*Pox B, indicating AHL degradation by the lactonase (Fig. 2A). Biofilm formation was assessed, but no biofilm formation was obtained in the tested conditions and no impact of *Sso*Pox treatment was observed (Fig. 2B). Swarming motility, a surface movement initiated by a group of bacteria and driven by rotating flagella (Kearns 2010), was evaluated. After 24 hours of incubation, untreated *B. glumae* were weakly motile and the zone surrounding the inoculation spot displayed a rounded shape. In contrast, bacteria grown on LB agar containing *Sso*Pox B showed increased motility, together with a change in colony pattern, the shape of which became irregular (Fig. S1, Supporting Information). Previous studies showed that motility regulation and efficiency is multifactorial and depends on QS, temperature, and the production of rhamnolipids, among other factors (Nickzad et al. 2015). Here, we report that AHL degradation at 30°C modulates swarming motility. As motility was associated with virulence and pathogenesis of *B. glumae* in rice (Kim et al. 2018), the impact of *Sso*Pox on the ability to infect plants was evaluated using a maceration assay on red onions. This infection model is an alternative to the rice infection model, and enables to assess the virulence of *B. glumae* with a high correlation between virulence in rice and onions (Karki et al. 2012). Onion slices were inoculated with *B. glumae* culture and measurements of the macerated area were performed. Onion slices infected with *B. glumae* were highly macerated (i.e. 64%), demonstrating the ability of *B. glumae* to infect onions (Fig. 2C). In contrast, the pretreatment of onion slices with *Sso*Pox B significantly inhibited the infection by *B. glumae* (*P-*value < .0001), decreasing the average maceration to 26%, corresponding to a 2.5-fold decrease. In summary, these results demonstrate that *Sso*Pox B degraded AHL produced by *B. glumae*, altered motility, and decreased maceration in an onion infection model. Previous studies using *B. glumae* strains expressing the *aii*A lactonase gene also reported a decrease in AHL production, as well as reduced virulence in rice (Cho et al. 2007, Park et al. 2010). Taken as a whole, these results suggest that QS inhibition decreases the degree of infections caused by *B. glumae* and that motility increase *in vitro* is not necessarily correlated with an increase in maceration symptoms and onion infections. Further investigations *in planta* are needed to confirm the potential of QQ as a biocontrol solution against this phytopathogen.

### *Sso*Pox activity against human pathogens

The ESKAPEE group designates pathogens known to escape drug therapies because of their multi resistance to antibiotics (Cieślik et al. 2021). The acronym ESKAPEE refers to *Enterococcus faecium, Staphylococcus aureus, Klebsiella pneumoniae, A. baumannii, P. aeruginosa, Enterobacter* species, and *E. coli*. These pathogens have been classified by the WHO in a priority list of antibiotic-resistant bacteria for which the development of new therapeutic solution is urgent (World Health Organization 2017). Among these species, several bacteria use a QS system to regulate their virulence (Galloway et al. 2012), and the disruption of this system of communication is studied as an alternative to antibiotic molecules (Nigam et al. 2014).

**Figure 3.**
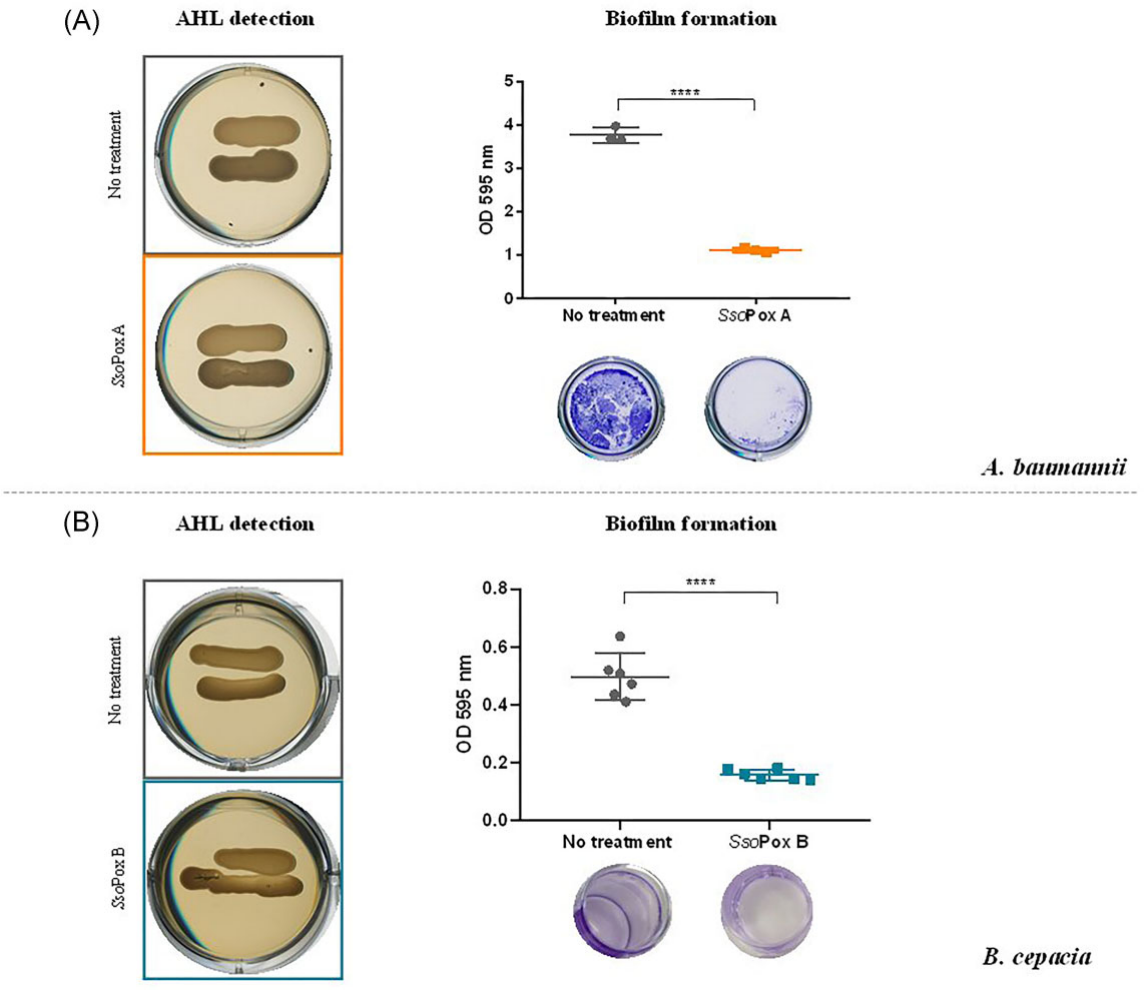
*Sso*Pox A decreases biofilm formation in human pathogenic bacteria. Detection of AHL production using CV026 and measurement of biofilm formation with crystal violet staining with (A) *A. baumannii*. Experiment performed in *n* = 3 replicates. ^****^P-value < .0001 according to Student’s *t*-test on GraphPad Prism 7.04. and with (B) *B. cepacia*. Experiment performed in *n* = 6 replicates. ^****^P-value < .0001 according to Student’s *t*-test on GraphPad Prism 7.04.

First, we tested the impact of QQ on *A. baumannii*. This Gram-negative bacterium causes a broad array of infections, including pneumonia, urinary tract infections, bloodstream infections, and skin infections, and has been found in many healthcare environments (Bhargava et al. 2010, Antunes et al. 2011). Several lactones were reported in *A. baumannii*, mainly long-chain AHLs varying from C_10_ to C_16_. 3-OH-C_12_-HSL have been further described as the main AHL in *A. baumannii* (Saipriya et al. 2020). AHLs have also been shown to influence biofilm formation which is an important virulence factor involved in antibiotic resistance and survival properties (Bhargava et al. 2010, Mayer et al. 2020). The *Sso*Pox A variant was used to assess its QQ activity against *A. baumannii*. As the kinetic parameters could not be performed on 3-OH-C_12_-HSL, which is not commercially available, the *Sso*Pox variant was chosen with respect to its activity against long-chain AHLs, including 3-oxo-C_12_-HSL (Table 3). Culture of *A. baumannii* failed to induce violacein production in the reporter strain CV026 (Fig. 3A). Nevertheless, an AHL synthase *abaI* was identified in the genome of the *A. baumannii* strain used in this study. In addition, *C. violaceum* CV026 is particularly adapted to the detection of AHLs from C_4_ to C_8_, and was shown not to produce violacein in the presence of long-chain AHLs, even at high concentrations (McClean et al. 1997). Therefore, biofilm formation was directly evaluated as a QS phenotype. After 48 hours of culture, a high biofilm formation was found in the untreated condition, whereas supplementation with *Sso*Pox A lactonase induced a 4-fold decrease in the quantity of biofilm (Fig. 3A). Similar results were obtained with *A. baumannii* ATCC 17978 and the lactonase Aii20J, significantly decreasing the formation of biofilm (Mayer et al. 2020), as well as in clinical isolates of *A. baumannii* (López et al. 2017), underlining the potential use of QQ as an alternative treatment to fight *A. baumannii* infections.

*Burkholderia cepacia*, another human pathogen, was then considered. *B. cepacia* is an important pathogen involved in cystic fibrosis (Geisenberger et al. 2000), known to produce AHLs. C_8_-HSL has been described as the main AHL, while the production of C_6_-HSL has also been reported (Riedel et al. 2001, Vial et al. 2007). Here, 24-hour cultures of *B. cepacia* treated or not treated with *Sso*Pox B were used for AHL detection, but no violacein production was observed in any of the samples, although the AHL synthase *cepI* was identified in the genome of the strain used for this assay (Fig. 3B). Therefore, biofilm formation was studied as a direct readout for QS and QQ using crystal violet staining, as previously described. In our conditions, the amount of biofilm was reduced upon treatment with *Sso*Pox B (Fig. 3B). Biofilm formation is a phenotype known to be under QS regulation in members of *B. cepacia complex*, such as *B. cepacia* H111 (Huber et al. 2001). Here, the results suggest that QQ may also influence biofilm formation in *B. cepacia* ATCC 25416 strain, although further investigations would be required to confirm QQ efficiency on other specific phenotypes.

**Figure 4.**
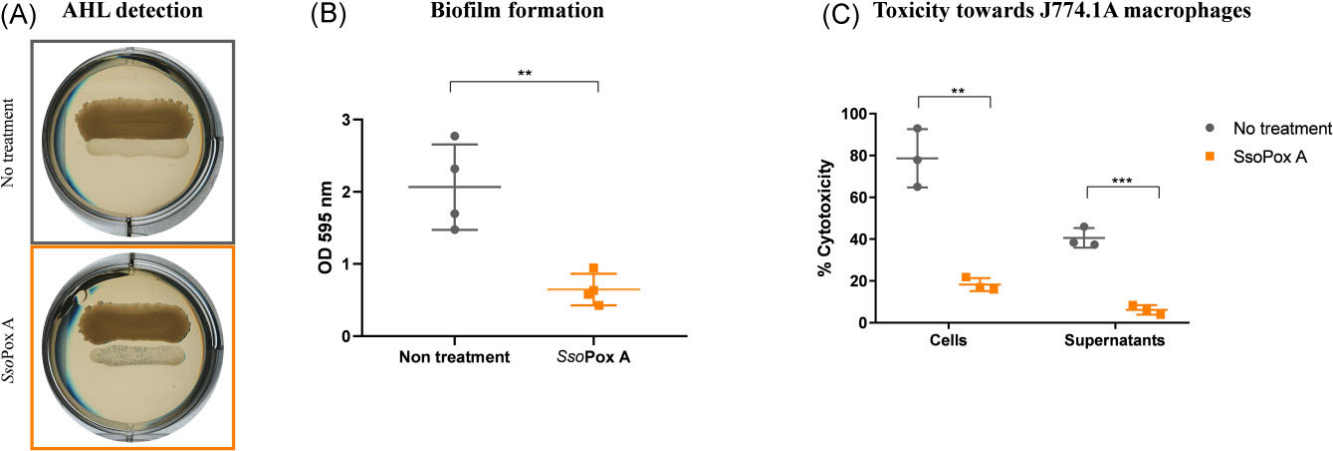
*Sso*Pox A decreases biofilm formation and cytotoxicity of *P. aeruginosa*. (A) Detection of AHL production using CV026. (B) Measurement of biofilm formation with crystal violet staining. Experiment performed in *n* = 3 replicates. ** *P*-value < .01 according to Student’s *t*-test on GraphPad Prism 7.04. (C) Cytotoxicity of *P. aeruginosa* cells and supernatants toward J774.1A macrophages with *Sso*Pox A treatment or not. Experiment was performed in *n* = 3 replicates. ** *P*-value < .01, *** *P*-value < .001 according to Student’s *t*-test on GraphPad Prism 7.04.

Finally, another human pathogen, *P. aeruginosa*, was tested. *Pseudomonas aeruginosa* is known to be responsible for hospital-acquired infections, particularly in immunocompromized patients and patients with cystic fibrosis (Eberl and Tümmler 2004, Palleroni 2010). The QS of *P. aeruginosa* PA14 involves two major AHL-based QS systems, the LasIR and RhlIR, which produce C_4_-HSL and 3-oxo-C_12_-HSL (Li et al. 2022). In this experimental set-up, no AHL was detected by the reporter strain *C. violaceum* CV026 (Fig. 4A), but the same reporter strain detected AHLs in ethyl acetate extractions of *P. aeruginosa* supernatants (Rémy et al. 2020). The addition of *Sso*Pox A, the variant selected for its degrading activity against 3-oxo-C_12_-HSL (Table 3), induced a 3-fold decrease in biofilm formation (Fig. 4B), confirming previous results obtained with *P. aeruginosa* PA14 and PAO1 (Hraiech et al. 2014, Guendouze et al. 2017, Rémy et al. 2020). The QQ activity of *Sso*Pox A was further evaluated in an *in vitro* cytotoxicity model using macrophages. The bacterial cytotoxicity at a MOI of 10 was assessed toward J774.1 macrophage cell lines. Untreated cultures of *P. aeruginosa* were strongly cytotoxic towards macrophages and 80% cytotoxicity was measured, while upon enzymatic treatment the cytotoxicity against macrophages decreased down to 20%, demonstrating the efficiency of QQ at reducing *P. aeruginosa* cytotoxicity and virulence factor production in this model. The cytotoxicity of cell-free supernatants was also evaluated. Cytotoxicity was detected in untreated supernatants, where 40% of cytotoxicity was measured, while cytotoxicity decreased below 10% in supernatants treated with *Sso*Pox A (Fig. 4C). These results demonstrate the impact of QS disruption by *Sso*Pox A treatment to tackle *P. aeruginosa* virulence in an *in vitro* model. *Pseudomonas aeruginosa* strains were largely used for QQE probing. Using this bacterial model, several studies have demonstrated that QQE may reduce various virulence factors such as motility, biofilm formation, and virulence against model organisms (*Caernorhabditis elegans, Drosophila melanogaster*, and mice) (Hraiech et al. 2014, Sikdar and Elias 2020). In addition, recent studies have demonstrated that the use of a QQ lactonase can significantly modulate *P. aeruginosa* PA14 susceptibility or resistance to antimicrobials (Rémy et al. 2020, Sikdar and Elias 2022). The synergistic effect of combining the QQE and antimicrobials (e.g. bacteriophages and antibiotics) to counteract *P. aeruginosa* PA14 virulence was also evaluated demonstrating the promising potential of this enzyme against human pathogenic strains (Mion et al. 2019).

### *Sso*Pox inhibits virulence factors in aquaculture fish pathogens

In aquaculture, a significant proportion of periodical disease outbreaks is due to bacterial pathogens (Dadar et al. 2017). Among these diseases, vibriosis and aeromonasis are of crucial interest in aquaculture (Jayaprakashvel and Subramani 2019). To treat these pathogens, vaccination, antibiotics, and bacteriophages are the most studied methods (Defoirdt et al. 2011, Richards 2014, Dadar et al. 2017), and QQ is a developing method, which is being considered to fight bacterial pathogens (Jayaprakashvel and Subramani 2019).

The fish pathogen *A. salmonicida*, was first tested. *Aeromonas sp*. are Gram-negative bacteria living ubiquitously in aquatic environments and are opportunistic pathogens of animals, mainly fish, and humans (Talagrand-Reboul et al. 2017, Vincent et al. 2019). *Aeromonas salmonicida* is responsible for furunculosis and septicaemia in fish (Tewari et al. 2014, Kim et al. 2015, Menanteau-Ledouble et al. 2016), and is the cause of severe economic losses in the aquaculture industry (Vincent et al. 2017). *Aeromonas salmonicida* uses two QS systems, the AI-2 and the AHL system, based on AsaIR (Swift et al. 1997, Jangid et al. 2007).

Previous studies have reported that the *A. salmonicida* strain ATCC 33658 mainly produces C_4_-HSL and C_6_-HSL, as confirmed by thin layer chromatography (Swift et al. 1997, Talagrand-Reboul et al. 2017), as well as C_8_-HSL, C_10_-HSL, C_12_-HSL, and C_14_-HSL, as detected by gas chromatography (Cataldi et al. 2007, Talagrand-Reboul et al. 2017).

**Figure 5.**
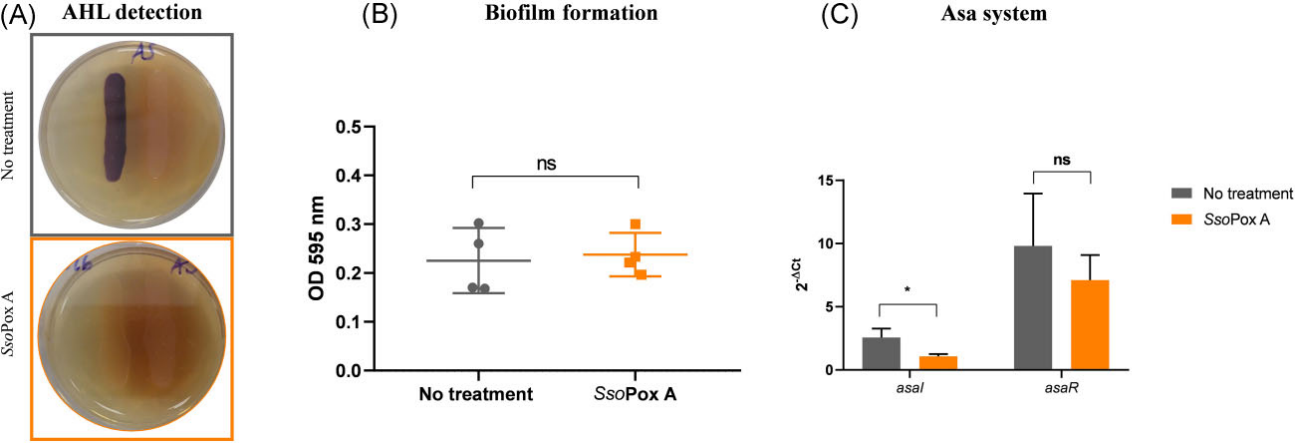
*Sso*Pox A alters QS of *A. salmonicida*. (A) Detection of AHL production using *C. violaceum* CV026. (B) Measurement of biofilm formation with crystal violet staining. ns: nonsignificant, according to Mann–Whitney test on GraphPad Prism 7.04. (C) Relative expression of genes involved in the Asa system after lactonase treatment. The experiment was performed in *n* = 6 replicates. ns: nonsignificant, *P-value < .05, according to Multiple *t*-tests on GraphPad Prism 7.04.

In this study, the impact of *Sso*Pox A was assessed focusing on AHL production, biofilm formation, and *asaRI* gene expression (Fig. 5). First, the production of AHLs by *A. salmonicida* was confirmed by the reporter strain *C. violaceum* CV026, while the addition of *Sso*Pox A resulted in the absence of violacein production, confirming its ability to degrade the corresponding AHLs (Fig. 5A). To study the impact of QS and QQ on *A. salmonicida* phenotypes, biofilm formation was then measured. Although *Aeromonas* strains are known to produce biofilm (Talagrand-Reboul et al. 2017), no biofilm was detected in our conditions (Fig. 5B). Therefore, the expression of the genes involved in *A. salmonicida* QS system was checked to evaluate the impact of the lactonase on the QS of the strain. The expression of *asaI* was significantly decreased in the presence of *Sso*Pox A, as compared to the control without the enzyme. However, the expression of the *asaR* gene was not significantly impacted by the presence of the enzyme in comparison to the control without the enzyme (Fig. 5C). These results show that *Sso*Pox is able to affect QS in *A. salmonicida* ATCC 33658. Indeed, AHL degradation by *Sso*Pox prevents AsaR activation by AHL binding, therefore limiting the induction of *asaI* expression. Further impact on *A. salmonicida* virulence remains to be studied but in two other *A. salmonicida* strains, the AsaIR system was correlated to virulence factor modulation. In *A. salmonicida* subsp. *achromogenes*, a *ΔasaI* mutant was not able to produce AHLs, and its virulence was decreased in Arctic charr (*Salvelinus alpinus* L.) (Schwenteit et al. 2011). In *A. salmonicida* AE03, also a *ΔasaI* mutant, the production of C_4_- and C_6_-HSL was abolished, while C_8_-, C_10_-, and C_12_-HSL were still detected and the maximum biofilm biomass and thickness were decreased (Liu et al. 2018).

**Figure 6.**
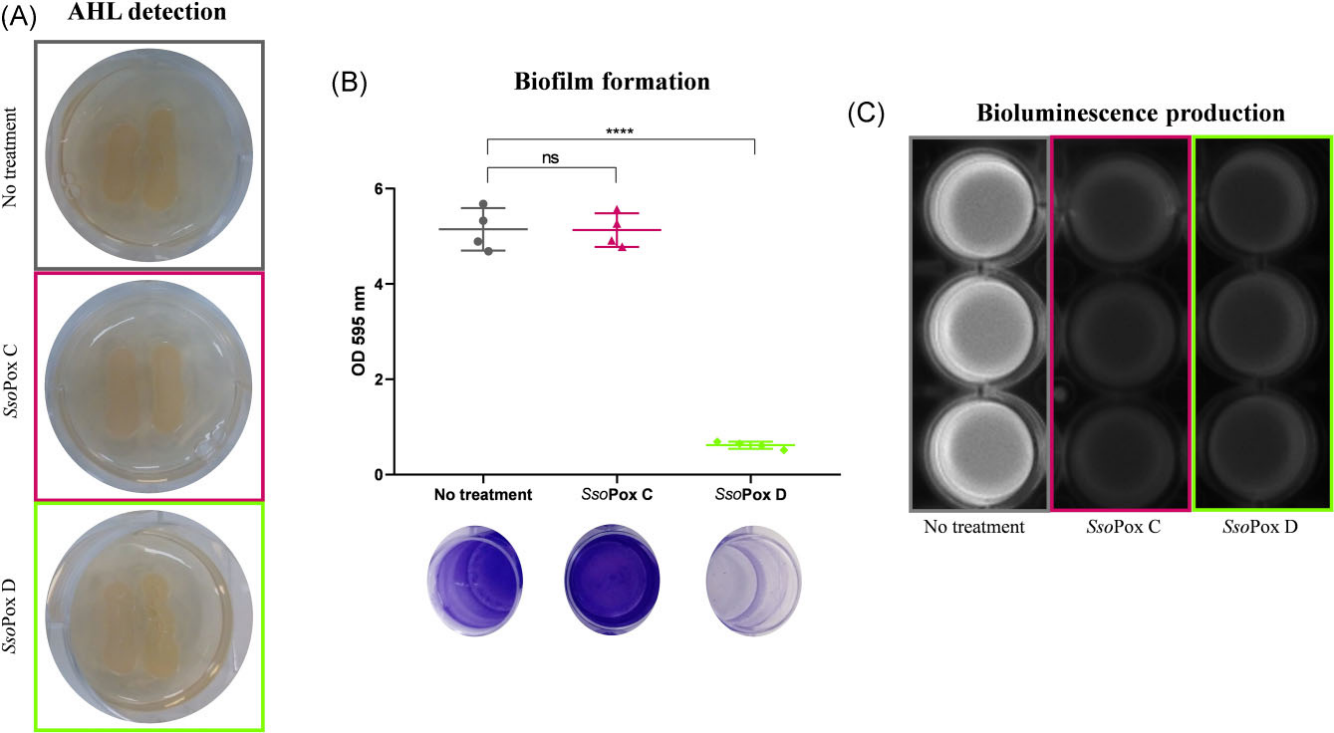
*Sso*Pox alters biofilm formation and bioluminescence in *V. harveyi*. (A) Detection of AHL production using *C. violaceum* CV026. (B) Measurement of biofilm formation with crystal violet staining. The experiment was performed in *n* = 4 replicates. ns: nonsignificant, ^****^P-value < .0001 according to Student’s *t*-test on GraphPad Prism 7.04. (C) Pictures of bioluminescence production.

A second marine gammaproteobacterium, *V. harveyi*, was then tested. *Vibrio harveyi* naturally lives in marine habitats, especially in warm waters and represents, together with other *Vibrio* species, a serious pathogen of wild and farmed fish and invertebrates (Zhang et al. 2020). Among its virulence arsenal, the productions of extracellular toxins, exoproteases, and siderophores (Defoirdt et al. 2008) are known to be regulated by QS. In addition to its ecological and economic significance, *V. harveyi* uses a QS system that differs from any other Gram-negative bacteria. This QS system relies on three AIs, including the AHL 3-OH-C_4_-HSL (HAI-1), that are sensed extracellularly by their cognate sensor kinase, and the QS signal is transmitted to the LuxR regulator via a phosphorelay cascade (Cao and Meighen 1989, Chen et al. 2002, Ng et al. 2011). Finally, *V. harveyi* is also known for its easy QS readout, bioluminescence, which is involved in the milky sea effect (Lapota et al. 1988, Miller et al. 2005). As no degradation signal was obtained with any *Sso*Pox variant using 3-OH-C_4_-HSL, no kinetic parameters could be determined. However, two variants identified for their ability to degrade AHLs with short acyl chains were selected, namely *Sso*Pox C and D (Table 3). Similarly, the reporter strain *C. violaceum* CV026 did not detect 3-OH-C_4_-HSL, so that no direct evidence for degradation of this AHL by the selected variants could be observed (Fig. 6A; Fig. S2, Supporting Information). Nevertheless, both variants successfully inhibited bioluminescence (Fig. 6C), indicating that QS could be disrupted by *Sso*Pox. In addition, QS disruption was checked using biofilm formation as a second QS biomarker. Interestingly, while biofilm formation was not at all impacted by the variant *Sso*Pox C, it was fully abolished using *Sso*Pox D (Fig. 6B), suggesting a major difference in the kinetics of both variants to degrade the AHL used by *V. harveyi*. Taken as a whole, these results indicate that QS can be interrupted even when only one of the signals is degraded, probably by destabilizing the timing of autoinducer production (Anetzberger et al. 2012), or by perturbing the phosphorylation flow and the feedback loops necessary to maintain the robustness of the phosphorelay cascade (Plener et al. 2015, Papenfort and Bassler 2016). In the context of global warming, the increased presence of *Vibrio* species in northern seas could be correlated with the increase in sea surface temperature (Vezzulli et al. 2016, Vezzulli 2022), increasing the risk of disease outbreaks in marine organisms that could spread to humans through infected water or seafood (Newton et al. 2012, Montánchez and Kaberdin 2020). Many potential vaccines have been developed in recent years against different *Vibrio* sp. but their use has thus far been limited to laboratories (Xu et al. 2022). To study the potential of QQ and particularly that of lactonases as a reliable strategy to limit antibiotic use against vibriosis, *in vivo* studies need to be performed (Tinh et al. 2007, Pande et al. 2013, Noor et al. 2019). Most studies are based on the use of bacterial mutants unable to produce and/or perceive QS signals. Therefore, the outcome might differ from QQ approaches that induce a metabolic sink by constantly degrading AHL produced by bacteria. Indeed, the addition of AHL-degrading bacteria (*Bacillus* sp.) protected giant river prawn larvae (*Macrobrachium rosenbergii*) against *V. campbellii* virulence (Pande et al. 2013), and the direct injection of a lactonase in *Carassius auratus* significantly protected goldfish against infection by *A. hydrophila* (Peng et al. 2021).

## Conclusion

Taken as a whole, these results show that lactonase-mediated QS interference is of utmost interest when it comes to targeting the virulence of bacterial pathogens. Although the different bacteria did not respond equally to lactonase treatments, all were nevertheless affected in at least one QS-related phenotype. Depending on the communication molecules used and the bacterial regulatory QS circuit, the very same lactonase can be engineered by specific point mutations to efficiently target a broad range of bacteria and tackle their virulence. *In vivo* studies are needed to reinforce the previous work and assess the effectiveness of *Sso*Pox treatment. To harness this broad-spectrum potential for a variety of applications, enzymes need to be competitive in comparison with conventional approaches, and enzyme engineering seems an attractive way to make this technology cost effective. Moreover, cautious attention must be paid to regulatory requirements, as optimized enzymes issued from genetically modified microorganisms must fit within current regulatory frameworks, such as international regulations on health products, veterinary medicinal products, and plant protection products. Although several aspects need to be considered before reaching industrial applications, this study suggests that lactonases could constitute a sustainable approach to address health threats in the animal–human–environmental interface. These results, combined with the high stability of *Sso*Pox, will pave the way for concrete applications and broaden the arsenal available to fight bacterial infections.

## Supplementary Material

ftae009_Supplemental_Files

## References

[bib1] Anetzberger C, Reiger M, Fekete A et al. Autoinducers act as biological timers in *Vibrio harveyi*. PLoS One. 2012;7:e48310. 23110227 10.1371/journal.pone.0048310 PMC3482212

[bib2] Antunes LCS, Imperi F, Carattoli A et al. Deciphering the multifactorial nature of *Acinetobacter baumannii* pathogenicity. In: Adler B (ed.), PLoS One. 2011;6:e22674. 21829642 10.1371/journal.pone.0022674 PMC3148234

[bib3] Barnard AML, Bowden SD, Burr T et al. Quorum sensing, virulence and secondary metabolite production in plant soft-rotting bacteria. Phil Trans R Soc B. 2007;362:1165–83. 17360277 10.1098/rstb.2007.2042 PMC2435580

[bib4] Bhargava N, Sharma P, Capalash N. Quorum sensing in *Acinetobacter*: an emerging pathogen. Crit Rev Microbiol. 2010;36:349–60. 20846031 10.3109/1040841X.2010.512269

[bib5] Billot R, Plener L, Grizard D et al. Applying molecular and phenotypic screening assays to identify efficient quorum quenching lactonases. Enzyme Microb Technol. 2022;160:110092. 35797848 10.1016/j.enzmictec.2022.110092 PMC10310055

[bib6] Billot R, Plener L, Jacquet P et al. Engineering acyl-homoserine lactone-interfering enzymes towards bacterial control. J Biol Chem. 2020;295:12993–3007. 32690609 10.1074/jbc.REV120.013531 PMC7489903

[bib7] Bradford MM . A rapid and sensitive method for the quantitation of microgram quantities of protein utilizing the principle of protein-dye binding. Anal Biochem. 1976;72:248–54. 942051 10.1016/0003-2697(76)90527-3

[bib8] Bzdrenga J, Daudé D, Rémy B et al. Biotechnological applications of quorum quenching enzymes. Chem Biol Interact. 2017;267:104–15. 27223408 10.1016/j.cbi.2016.05.028

[bib9] Cao JG, Meighen EA. Purification and structural identification of an autoinducer for the luminescence system of *Vibrio harveyi*. J Biol Chem. 1989;264:21670–6. 2600086

[bib10] Carstens A, Djurhuus A, Kot W et al. Unlocking the potential of 46 new bacteriophages for biocontrol of *Dickeya Solani*. Viruses. 2018;10:621. 30423804 10.3390/v10110621 PMC6267328

[bib11] Cataldi TRI, Bianco G, Palazzo L et al. Occurrence of N-acyl-l-homoserine lactones in extracts of some Gram-negative bacteria evaluated by gas chromatography–mass spectrometry. Anal Biochem. 2007;361:226–35. 17207763 10.1016/j.ab.2006.11.037

[bib12] Charkowski AO . The changing face of bacterial soft-rot diseases. Annu Rev Phytopathol. 2018;56:269–88. 29958075 10.1146/annurev-phyto-080417-045906

[bib13] Chen X, Schauder S, Potier N et al. Structural identification of a bacterial quorum-sensing signal containing boron. Nature. 2002;415:545–9. 11823863 10.1038/415545a

[bib14] Cho H-S, Park S-Y, Ryu C-M et al. Interference of quorum sensing and virulence of the rice pathogen *Burkholderia glumae* by an engineered endophytic bacterium: biocontrol of *B. glumae* by an engineered endophyte. FEMS Microbiol Ecol. 2007;60:14–23. 17313662 10.1111/j.1574-6941.2007.00280.x

[bib15] Choi O, Kim S, Kang B et al. Genetic diversity and distribution of Korean isolates of *Burkholderia glumae*. Plant Dis. 2021;105:1398–407. 33325743 10.1094/PDIS-08-20-1795-RE

[bib16] Cieślik M, Bagińska N, Górski A et al. Animal models in the evaluation of the effectiveness of phage therapy for infections caused by Gram-negative bacteria from the ESKAPE group and the reliability of its use in humans. Microorganisms. 2021;9:206. 33498243 10.3390/microorganisms9020206 PMC7909267

[bib17] Crépin A, Barbey C, Beury-Cirou A et al. Quorum sensing signaling molecules produced by reference and emerging soft-Rot bacteria (*Dickeya* and *Pectobacterium* spp.). PLoS One. 2012;7:e35176. 22539957 10.1371/journal.pone.0035176 PMC3335102

[bib18] Cui G, Yin K, Lin N et al. *Burkholderia gladioli* CGB10: a novel strain biocontrolling the sugarcane smut disease. Microorganisms. 2020;8:1943. 33297590 10.3390/microorganisms8121943 PMC7762381

[bib19] Cui Z, Wang S, Kakar KU et al. Genome sequence and adaptation analysis of the Human and rice pathogenic strain *Burkholderia glumae* AU6208. Pathogens. 2021;10:87. 33498266 10.3390/pathogens10020087 PMC7909282

[bib20] Dadar M, Dhama K, Vakharia VN et al. Advances in aquaculture vaccines against fish pathogens: global status and current trends. Rev Fish Sci Aquacult. 2017;25:184–217.

[bib21] Daude D, Elias M, Chabriere E et al. Novel Mutated Lactonase Enzymes, Compositions Containing Them and Uses Thereof. Geneva: WIPO, 2023.

[bib22] Defoirdt T, Boon N, Sorgeloos P et al. Quorum sensing and quorum quenching in *Vibrio harveyi*: lessons learned from in vivo work. ISME J. 2008;2:19–26. 18180744 10.1038/ismej.2007.92

[bib23] Defoirdt T, Sorgeloos P, Bossier P. Alternatives to antibiotics for the control of bacterial disease in aquaculture. Curr Opin Microbiol. 2011;14:251–8. 21489864 10.1016/j.mib.2011.03.004

[bib24] Dong Y-H, Wang L-H, Xu J-L et al. Quenching quorum-sensing-dependent bacterial infection by an N-acyl homoserine lactonase. Nature. 2001;411:813–7. 11459062 10.1038/35081101

[bib25] Eberl L, Tümmler B. *Pseudomonas aeruginosa* and *Burkholderia cepacia* in cystic fibrosis: genome evolution, interactions and adaptation. Int J Med Microbiol. 2004;294:123–31. 15493822 10.1016/j.ijmm.2004.06.022

[bib26] Galloway WRJD, Hodgkinson JT, Bowden S et al. Applications of small molecule activators and inhibitors of quorum sensing in Gram-negative bacteria. Trends Microbiol. 2012;20:449–58. 22771187 10.1016/j.tim.2012.06.003

[bib27] Geisenberger O, Givskov M, Riedel K et al. Production of N-acyl-L-homoserine lactones by *P. aeruginosa* isolates from chronic lung infections associated with cystic fibrosis. FEMS Microbiol Lett. 2000;184:273–8. 10713433 10.1111/j.1574-6968.2000.tb09026.x

[bib28] Greenberg EP, Hastings JW, Ulitzur S. Induction of luciferase synthesis in *Beneckea harveyi* by other marine bacteria. Arch Microbiol. 1979;120:87–91.

[bib29] Guendouze A, Plener L, Bzdrenga J et al. Effect of quorum quenching lactonase in clinical isolates of *Pseudomonas aeruginosa* and comparison with quorum sensing inhibitors. Front Microbiol. 2017;08:1–10. 10.3389/fmicb.2017.00227 PMC5306132 28261183

[bib30] Ham JH, Melanson RA, Rush MC. *Burkholderia glumae*: next major pathogen of rice?. Mol Plant Pathol. 2011;12:329–39. 21453428 10.1111/j.1364-3703.2010.00676.x PMC6640401

[bib31] Harbarth S, Balkhy HH, Goossens H et al. Antimicrobial resistance: one world, one fight!. Antimicrob Resist Infect Control. 2015;4:49.

[bib32] Helman Y, Chernin L. Silencing the mob: disrupting quorum sensing as a means to fight plant disease. Mol Plant Pathol. 2015;16:316–29. 25113857 10.1111/mpp.12180 PMC6638422

[bib33] Hiblot J, Gotthard G, Elias M et al. Differential active site loop conformations mediate promiscuous activities in the lactonase SsoPox. PLoS One. 2013;8:1–14. 10.1371/journal.pone.0075272 PMC3781021 24086491

[bib34] Hoffman LR, D'Argenio DA, MacCoss MJ et al. Aminoglycoside antibiotics induce bacterial biofilm formation. Nature. 2005;436:1171–5. 16121184 10.1038/nature03912

[bib35] Hosseinzadeh S, Shams-Bakhsh M, Sadeghizadeh M. Attenuation and quantitation of virulence gene expression in quorum-quenched *Dickeya chrysanthemi*. Arch Microbiol. 2017;199:51–61. 27496158 10.1007/s00203-016-1276-7

[bib36] Hraiech S, Hiblot J, Lafleur J et al. Inhaled lactonase reduces *Pseudomonas aeruginosa* quorum sensing and mortality in rat pneumonia. In: Meijler MM (ed.), PLoS One. 2014;9:e107125. 25350373 10.1371/journal.pone.0107125 PMC4211673

[bib37] Huber B, Riedel K, Hentzer M et al. The cep quorum-sensing system of *Burkholderia cepacia* H111 controls biofilm formation and swarming motility. Microbiology. 2001;147:2517–28. 11535791 10.1099/00221287-147-9-2517

[bib38] Jangid K, Kong R, Patole MS et al. luxRI homologs are universally present in the genus *Aeromonas*. BMC Microbiol. 2007;7:93. 17953777 10.1186/1471-2180-7-93 PMC2180181

[bib39] Jayaprakashvel M, Subramani R. Implications of quorum sensing and quorum quenching in aquaculture health management. In: Bramhachari PV (ed.), Implication of Quorum Sensing and Biofilm Formation in Medicine, Agriculture and Food Industry. Singapore: Springer, 2019, 299–312.

[bib40] Jeong Y, Kim J, Kim S et al. Toxoflavin produced by *Burkholderia glumae* causing rice grain Rot is responsible for inducing bacterial wilt in many field crops. Plant Dis. 2003;87:890–5. 30812790 10.1094/PDIS.2003.87.8.890

[bib41] Joshi JR, Burdman S, Lipsky A et al. Effects of plant antimicrobial phenolic compounds on virulence of the genus *Pectobacterium*. Res Microbiol. 2015;166:535–45. 25981538 10.1016/j.resmic.2015.04.004

[bib42] Karki HS, Shrestha BK, Han JW et al. Diversities in virulence, antifungal activity, pigmentation and DNA fingerprint among strains of *Burkholderia glumae*. PLoS One. 2012;7:e45376. 23028972 10.1371/journal.pone.0045376 PMC3445519

[bib43] Kearns DB . A field guide to bacterial swarming motility. Nat Rev Micro. 2010;8:634–44. 10.1038/nrmicro2405 PMC3135019 20694026

[bib44] Khardori N, Stevaux C, Ripley K. Antibiotics: from the beginning to the future: part 2. Ind J Pediatr. 2020;87:43–47. 10.1007/s12098-019-03113-0 31808125

[bib45] Kim J, Kim J-G, Kang Y et al. Quorum sensing and the LysR-type transcriptional activator ToxR regulate toxoflavin biosynthesis and transport in *Burkholderia glumae*. Mol Microbiol. 2004;54:921–34. 15522077 10.1111/j.1365-2958.2004.04338.x

[bib46] Kim J, Mannaa M, Kim N et al. The roles of two hfq genes in the virulence and stress resistance of *Burkholderia glumae*. Plant Pathol J. 2018;34:412–25. 30369851 10.5423/PPJ.OA.06.2018.0097 PMC6200039

[bib47] Kim JH, Choresca CH, Shin SP et al. Biological control of *Aeromonas salmonicida* subsp. *salmonicida* infection in rainbow trout (*Oncorhynchus mykiss*) using *Aeromonas* phage PAS-1. Transbound Emerg Dis. 2015;62:81–86. 23594036 10.1111/tbed.12088

[bib48] Koch G, Nadal-Jimenez P, Reis CR et al. Reducing virulence of the human pathogen *Burkholderia* by altering the substrate specificity of the quorum-quenching acylase PvdQ. Proc Natl Acad Sci USA. 2014;111:1568–73. 24474783 10.1073/pnas.1311263111 PMC3910591

[bib49] Kusada H, Tamaki H, Kamagata Y et al. A novel quorum-quenching N-acylhomoserine lactone acylase from *Acidovorax* sp. strain MR-S7 mediates antibiotic resistance. Appl Environ Microb. 2017;83:e00080–17. 10.1128/AEM.00080-17 PMC5478981 28455333

[bib50] Lapota D, Galt C, Losee JR et al. Observations and measurements of planktonic bioluminescence in and around a milky sea. J Exp Mar Biol Ecol. 1988;119:55–81.

[bib51] Li Q, Mao S, Wang H et al. The molecular architecture of *Pseudomonas aeruginosa* quorum-sensing inhibitors. Mar Drugs. 2022;20:488. 36005489 10.3390/md20080488 PMC9409833

[bib52] Liu H, Coulthurst SJ, Pritchard L et al. Quorum sensing coordinates brute force and stealth modes of infection in the plant pathogen *Pectobacterium atrosepticum*. PLoS Pathog. 2008;4:e1000093. 10.1371/journal.ppat.1000093. 18566662 PMC2413422

[bib53] Liu L, Yan Y, Feng L et al. Quorum sensing asaI mutants affect spoilage phenotypes, motility, and biofilm formation in a marine fish isolate of *Aeromonas salmonicida*. Food Microbiol. 2018;76:40–51. 30166167 10.1016/j.fm.2018.04.009

[bib54] López M, Mayer C, Fernández-García L et al. Quorum sensing network in clinical strains of *A. baumannii*: aidA is a new quorum quenching enzyme. In: Gao F (ed.), PLoS One. 2017;12:e0174454. 28328989 10.1371/journal.pone.0174454 PMC5362224

[bib57] McClean KH, Winson MK, Fish L et al. Quorum sensing and *Chromobacterium violaceum*: exploitation of violacein production and inhibition for the detection of N-acylhomoserine lactones. Microbiology. 1997;143:3703–11. 9421896 10.1099/00221287-143-12-3703

[bib58] McEwen SA, Collignon PJ. Antimicrobial resistance: a One Health perspective. In: Antimicrobial Resistance in Bacteria from Livestock and Companion Animals. Hoboken: John Wiley & Sons, Ltd, 2018, 521–47.

[bib55] Mansfield J, Genin S, Magori S et al. Top 10 plant pathogenic bacteria in molecular plant pathology. Mol Plant Pathol. 2012;13:614–29. 22672649 10.1111/j.1364-3703.2012.00804.x PMC6638704

[bib56] Mayer C, Muras A, Parga A et al. Quorum sensing as a target for controlling surface associated motility and biofilm formation in *Acinetobacter baumannii* ATCC® 17978TM. Front Microbiol. 2020;11:565548. 33101239 10.3389/fmicb.2020.565548 PMC7554515

[bib59] Mee-Ngan Y, Yang C-H, Barak JD et al. The *Erwinia chrysanthemi* type III secretion system is required for multicellular behavior. J Bacteriol. 2005;187:639–48. 15629935 10.1128/JB.187.2.639-648.2005 PMC543537

[bib60] Menanteau-Ledouble S, Kumar G, Saleh M et al. *Aeromonas salmonicida*: updates on an old acquaintance. Dis Aquat Org. 2016;120:49–68. 10.3354/dao03006 27304870

[bib61] Meyer JM . The florescent pigment of *Pseudomonas fluorescens* biosynthesis, purification and physicalchemical properties. J Gen Microbiol. 1978;107:319–28.

[bib62] Miller SD, Haddock SHD, Elvidge CD et al. Detection of a bioluminescent milky sea from space. Proc Natl Acad Sci USA. 2005;102:14181–4. 16186481 10.1073/pnas.0507253102 PMC1242338

[bib63] Mion S, Carriot N, Lopez J et al. Disrupting quorum sensing alters social interactions in *Chromobacterium violaceum*. NPJ Biofilms Microbiomes. 2021;7:40. 33888726 10.1038/s41522-021-00211-w PMC8062528

[bib64] Mion S, Rémy B, Plener L et al. Quorum quenching lactonase strengthens bacteriophage and antibiotic arsenal against *Pseudomonas aeruginosa* clinical isolates. Front Microbiol. 2019;10. 10.3389/fmicb.2019.02049. PMC6734170 31551983

[bib65] Mizobuchi R, Fukuoka S, Tsuiki C et al. Evaluation of major rice cultivars for resistance to bacterial seedling rot caused by *Burkholderia glumae* and identification of Japanese standard cultivars for resistance assessments. Breed Sci. 2020;70:221–30. 32523404 10.1270/jsbbs.19117 PMC7272248

[bib66] Montánchez I, Kaberdin VR. *Vibrio harveyi*: a brief survey of general characteristics and recent epidemiological traits associated with climate change. Mar Environ Res. 2020;154:104850. 32056705 10.1016/j.marenvres.2019.104850

[bib67] Murugayah SA, Gerth ML. Engineering quorum quenching enzymes: progress and perspectives. Biochm Soc Trans. 2019:47:BST20180165. 10.1042/BST20180165 PMC6599154 31064863

[bib68] Nasser W, Dorel C, Wawrzyniak J et al. VFM a new quorum sensing system controls the virulence of *Dickeya dadantii*. Environ Microbiol. 2013;15:865–80. 23227918 10.1111/1462-2920.12049

[bib69] Newton A, Kendall M, Vugia DJ et al. Increasing rates of vibriosis in the United States, 1996–2010: review of surveillance data from 2 systems. Clin Infect Dis. 2012;54:S391–5. 22572659 10.1093/cid/cis243 PMC4604744

[bib70] Ng W-L, Perez LJ, Wei Y et al. Signal production and detection specificity in Vibrio CqsA/CqsS quorum-sensing systems. Mol Microbiol. 2011;79:1407–17. 21219472 10.1111/j.1365-2958.2011.07548.x PMC3285556

[bib71] Nickzad A, Lépine F, Déziel E. Quorum sensing controls swarming motility of *Burkholderia glumae* through regulation of rhamnolipids. PLoS One. 2015;10:e0128509. 26047513 10.1371/journal.pone.0128509 PMC4457897

[bib72] Nigam A, Gupta D, Sharma A. Treatment of infectious disease: beyond antibiotics. Microbiol Res. 2014;169:643–51. 24661689 10.1016/j.micres.2014.02.009

[bib73] Noor NM, Defoirdt T, Alipiah N et al. Quorum sensing is required for full virulence of *Vibrio campbellii* towards tiger grouper (*Epinephelus fuscoguttatus*) larvae. J Fish Dis. 2019;42:489–95. 30742313 10.1111/jfd.12946

[bib74] One Health . https://www.who.int/europe/initiatives/one-health (14 May 2024, date last accessed).

[bib75] Palleroni NJ . The *Pseudomonas* story. Environ Microbiol. 2010;12:1377–83. 20553550 10.1111/j.1462-2920.2009.02041.x

[bib76] Palmer AG, Streng E, Jewell KA et al. Quorum sensing in bacterial species that use degenerate autoinducers can be tuned by using structurally identical non-native ligands. ChemBioChem. 2011;12:138–47. 21154995 10.1002/cbic.201000551 PMC3181108

[bib77] Pande GSJ, Natrah FMI, Sorgeloos P et al. The *Vibrio campbellii* quorum sensing signals have a different impact on virulence of the bacterium towards different crustacean hosts. Vet Microbiol. 2013;167:540–5. 24055027 10.1016/j.vetmic.2013.08.021

[bib78] Papenfort K, Bassler BL. Quorum sensing signal-response systems in Gram-negative bacteria. Nat Rev Micro. 2016;14:576–88. 10.1038/nrmicro.2016.89 PMC5056591 27510864

[bib79] Park JY, Lee YH, Yang KY et al. AiiA-mediated quorum quenching does not affect virulence or toxoflavin expression in *Burkholderia glumae* SL2376: quorum-quenching effect on pathogenicity of *B. glumae*. Lett Appl Microbiol. 2010;51:619–24. 21039666 10.1111/j.1472-765X.2010.02940.x

[bib80] Peng M, Tong W, Zhao Z et al. Attenuation of *Aeromonas hydrophila* infection in *Carassius auratus* by YtnP, a N-acyl homoserine lactonase from *Bacillus licheniformis* T-1. Antibiotics. 2021;10:631. 34073161 10.3390/antibiotics10060631 PMC8228444

[bib81] Plener L, Lorenz N, Reiger M et al. The phosphorylation flow of the *Vibrio harveyi* quorum-sensing cascade determines levels of phenotypic heterogeneity in the population. J Bacteriol. 2015;197:1747–56. 25755191 10.1128/JB.02544-14 PMC4402392

[bib82] Rémy B, Mion S, Plener L et al. Interference in bacterial quorum sensing: a biopharmaceutical perspective. Front Pharmacol. 2018;9:203. 29563876 10.3389/fphar.2018.00203 PMC5845960

[bib83] Rémy B, Plener L, Decloquement P et al. Lactonase specificity is key to quorum quenching in *Pseudomonas aeruginosa*. Front Microbiol. 2020;11:762. 32390993 10.3389/fmicb.2020.00762 PMC7193897

[bib84] Rémy B, Plener L, Elias M et al. Des enzymes pour bloquer la communication bactérienne, une alternative aux antibiotiques?. Annales Pharmaceutiques Françaises. 2016;74:413–20. 27475310 10.1016/j.pharma.2016.06.005

[bib85] Richards GP . Bacteriophage remediation of bacterial pathogens in aquaculture: a review of the technology. Bacteriophage. 2014;4:e975540. 26713223 10.4161/21597081.2014.975540 PMC4590005

[bib86] Riedel K, Hentzer M, Geisenberger O et al. N-acylhomoserine-lactone-mediated communication between *Pseudomonas aeruginosa* and *Burkholderia cepacia* in mixed biofilms. Microbiology. 2001;147:3249–62. 11739757 10.1099/00221287-147-12-3249

[bib87] Saipriya K, Swathi CH, Ratnakar KS et al. Quorum-sensing system in *Acinetobacter baumannii*: a potential target for new drug development. J Appl Microbiol. 2020;128:15–27. 31102552 10.1111/jam.14330

[bib88] Schmittgen TD, Livak KJ. Analyzing real-time PCR data by the comparative C(T) method. Nat Protoc. 2008;3:1101–8. 18546601 10.1038/nprot.2008.73

[bib89] Schwenteit J, Gram L, Nielsen KF et al. Quorum sensing in *Aeromonas salmonicida* subsp. *achromogenes* and the effect of the autoinducer synthase AsaI on bacterial virulence. Vet Microbiol. 2011;147:389–97. 20708354 10.1016/j.vetmic.2010.07.020

[bib90] Shew AM, Durand-Morat A, Nalley LL et al. Warming increases bacterial panicle blight (*Burkholderia glumae*) occurrences and impacts on USA rice production. PLoS One. 2019;14:e0219199. 31295286 10.1371/journal.pone.0219199 PMC6623956

[bib91] Sikdar R, Elias M. Quorum quenching enzymes and their effects on virulence, biofilm, and microbiomes: a review of recent advances. Expert Rev Anti Infect Ther. 2020;18:1221–33. 32749905 10.1080/14787210.2020.1794815 PMC7705441

[bib92] Sikdar R, Elias MH. Evidence for complex interplay between quorum sensing and antibiotic resistance in *Pseudomonas aeruginosa*. Microbiol Spectr. 2022;10:e0126922. 36314960 10.1128/spectrum.01269-22 PMC9769976

[bib93] Smadja B, Latour X, Faure D et al. Involvement of *N*-acylhomoserine lactones throughout plant infection by *Erwinia carotovora* subsp. Atroseptica (*Pectobacterium atrosepticum*). MPMI. 2004;17:1269–78. 15553252 10.1094/MPMI.2004.17.11.1269

[bib94] Starr MP, Chatterjee AK, Starr PB et al. Enzymatic degradation of polygalacturonic acid by *Yersinia* and *Klebsiella* species in relation to clinical laboratory procedures. J Clin Microbiol. 1977;6:379–86. 334794 10.1128/jcm.6.4.379-386.1977 PMC274778

[bib95] Swift S, Karlyshev AV, Fish L et al. Quorum sensing in *Aeromonas hydrophila* and *Aeromonas salmonicida*: identification of the LuxRI homologs AhyRI and AsaRI and their cognate N-acylhomoserine lactone signal molecules. J Bacteriol. 1997;179:5271–81. 9286976 10.1128/jb.179.17.5271-5281.1997 PMC179392

[bib96] Talagrand-Reboul E, Jumas-Bilak E, Lamy B. The social life of *Aeromonas* through biofilm and quorum sensing systems. Front Microbiol. 2017;8. 10.3389/fmicb.2017.00037. PMC5247445 28163702

[bib97] Tewari R, Dudeja M, Nandy S et al. Isolation of *Aeromonas salmonicida* from human blood sample: a case report. J Clin Diagnost Res. 2014;8:139. 10.7860/JCDR/2014/6883.4032 PMC3972533 24701507

[bib98] Tinh NTN, Linh ND, Wood TK et al. Interference with the quorum sensing systems in a *Vibrio harveyi* strain alters the growth rate of gnotobiotically cultured rotifer *Brachionus plicatilis*. J Appl Microbiol. 2007;103:194–203. 17584465 10.1111/j.1365-2672.2006.03217.x

[bib99] Toth IK, Bell KS, Holeva MC et al. Soft rot erwiniae: from genes to genomes. Mol Plant Pathol. 2003;4:17–30. 20569359 10.1046/j.1364-3703.2003.00149.x

[bib100] Vezzulli L . Global expansion of *Vibrio* spp. in hot water. Environ Microbiol Rep. 2023;15:77–79. 36519781 10.1111/1758-2229.13135 PMC10103853

[bib101] Vezzulli L, Grande C, Reid PC et al. Climate influence on *Vibrio* and associated human diseases during the past half-century in the coastal North Atlantic. Proc Natl Acad Sci USA. 2016;113:E5062–5071. 27503882 10.1073/pnas.1609157113 PMC5003230

[bib102] Vial L, Groleau M-C, Dekimpe V et al. *Burkholderia* diversity and versatility: an inventory of the extracellular products. J Microbiol Biotechnol. 2007;17:1407–29. 18062218

[bib103] Vincent AT, Fernández-Bravo A, Sanchis M et al. Investigation of the virulence and genomics of *Aeromonas salmonicida* strains isolated from human patients. Infect Genet Evol. 2019;68:1–9. 30502493 10.1016/j.meegid.2018.11.019

[bib104] Vincent AT, Paquet VE, Bernatchez A et al. Characterization and diversity of phages infecting *Aeromonas salmonicida* subsp. *salmonicida*. Sci Rep. 2017;7:7054. 28765570 10.1038/s41598-017-07401-7 PMC5539321

[bib105] World Health Organization . Global Action Plan on Antimicrobial Resistance. Geneva: World Health Organization, 2015. 10.7196/samj.9644 26242647

[bib106] World Health Organization . WHO Publishes List of Bacteria for Which New Antibiotics Are Urgently Needed. Geneva: World Health Organization, 2017.

[bib107] Xu K, Wang Y, Yang W et al. Strategies for prevention and control of vibriosis in Asian fish culture. Vaccines. 2022;11:98. 36679943 10.3390/vaccines11010098 PMC9862775

[bib108] Xu Z, Liang Y, Lin S et al. Crystal violet and XTT assays on *Staphylococcus aureus* biofilm quantification. Curr Microbiol. 2016;73:474–82. 27324342 10.1007/s00284-016-1081-1

[bib109] Zhang X-H, He X, Austin B. *Vibrio harveyi*: a serious pathogen of fish and invertebrates in mariculture. Mar Life Sci Technol. 2020;2:231–45. 32419972 10.1007/s42995-020-00037-z PMC7223180

