# Supplementary material for: Disrupting quorum sensing as a strategy to inhibit bacterial virulence in human, animal, and plant pathogens
Source: Pathog Dis. 2024 May 9;82:ftae009. doi: 10.1093/femspd/ftae009 (PMC11110857; doi:10.1093/femspd/ftae009)
Supplement: ftae009_Supplemental_Files [file ftae009_supplemental_files.zip › Supplementary data Legends.docx]

**Figure S1.** *Sso*Pox treatment increases the swarming motility of *B. glumae.* Motility area was measured using ImageJ tool. ***p*-value <0.01 according to Student’s *t*-test on GraphPad Prism 7.04.

**Figure S2.** Detection of AHLs by the reporter strain *C. violaceum* CV026 using commercial AHLs with short- to long-acyl side chains.
