# Supplementary figures and images for: Disrupting quorum sensing as a strategy to inhibit bacterial virulence in human, animal, and plant pathogens
Source: Pathog Dis. 2024 May 9;82:ftae009. doi: 10.1093/femspd/ftae009 (PMC11110857; doi:10.1093/femspd/ftae009)

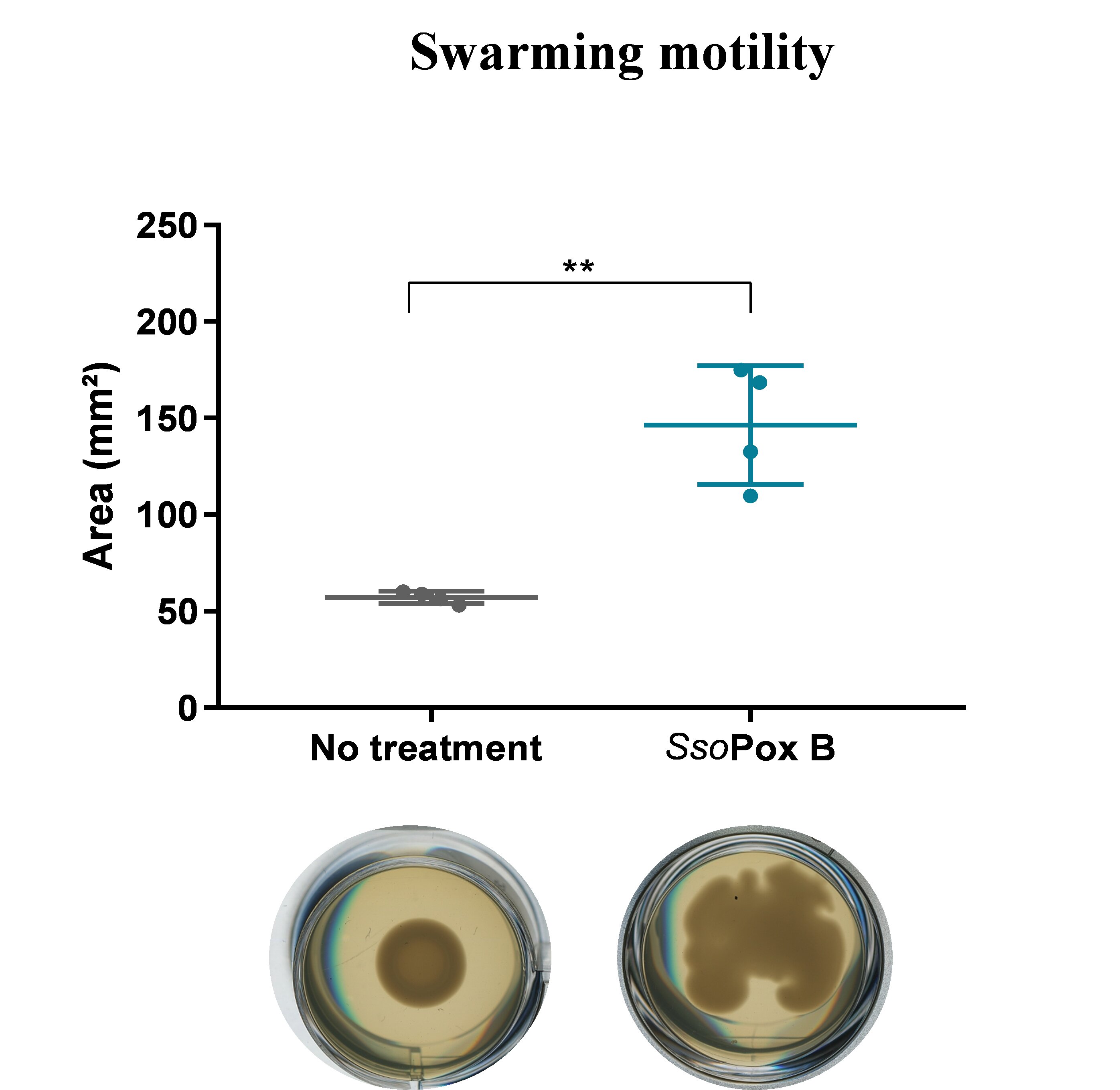

Supplement: ftae009_Supplemental_Files [file ftae009_supplemental_files.zip › FIGURE S1.jpg]

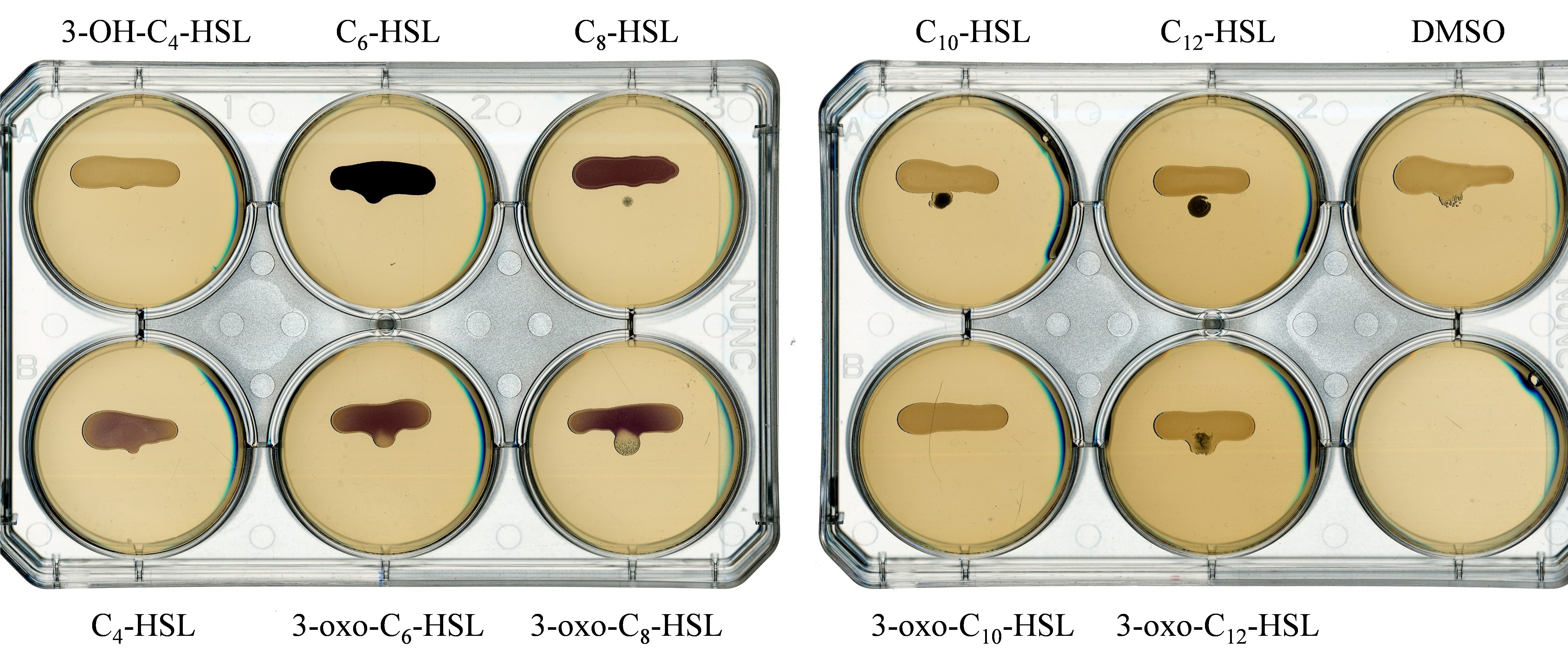

Supplement: ftae009_Supplemental_Files [file ftae009_supplemental_files.zip › FIGURE S2.jpg]
